# Convolutional Neural Network Techniques for Brain Tumor Classification (from 2015 to 2022): Review, Challenges, and Future Perspectives

**DOI:** 10.3390/diagnostics12081850

**Published:** 2022-07-31

**Authors:** Yuting Xie, Fulvio Zaccagna, Leonardo Rundo, Claudia Testa, Raffaele Agati, Raffaele Lodi, David Neil Manners, Caterina Tonon

**Affiliations:** 1 Department of Biomedical and Neuromotor Sciences, University of Bologna, 40126 Bologna, Italy; yuting.xie2@unibo.it (Y.X.); fulvio.zaccagna@unibo.it (F.Z.); raffaele.lodi@unibo.it (R.L.); caterina.tonon@unibo.it (C.T.); 2 Functional and Molecular Neuroimaging Unit, IRCCS Istituto delle Scienze Neurologiche di Bologna, Bellaria Hospital, 40139 Bologna, Italy; claudia.testa@unibo.it; 3 Department of Information and Electrical Engineering and Applied Mathematics, University of Salerno, 84084 Fisciano, Italy; lrundo@unisa.it; 4 Department of Physics and Astronomy, University of Bologna, 40127 Bologna, Italy; 5 Programma Neuroradiologia con Tecniche ad elevata complessità, IRCCS Istituto delle Scienze Neurologiche di Bologna, Bellaria Hospital, 40139 Bologna, Italy; raffaele.agati@isnb.it; 6 IRCCS Istituto delle Scienze Neurologiche di Bologna, Bellaria Hospital, 40139 Bologna, Italy

**Keywords:** deep learning, convolutional neural network, brain tumor classification, magnetic resonance imaging, clinical application, clinical effectiveness, computer-aided diagnosis

## Abstract

Convolutional neural networks (CNNs) constitute a widely used deep learning approach that has frequently been applied to the problem of brain tumor diagnosis. Such techniques still face some critical challenges in moving towards clinic application. The main objective of this work is to present a comprehensive review of studies using CNN architectures to classify brain tumors using MR images with the aim of identifying useful strategies for and possible impediments in the development of this technology. Relevant articles were identified using a predefined, systematic procedure. For each article, data were extracted regarding training data, target problems, the network architecture, validation methods, and the reported quantitative performance criteria. The clinical relevance of the studies was then evaluated to identify limitations by considering the merits of convolutional neural networks and the remaining challenges that need to be solved to promote the clinical application and development of CNN algorithms. Finally, possible directions for future research are discussed for researchers in the biomedical and machine learning communities. A total of 83 studies were identified and reviewed. They differed in terms of the precise classification problem targeted and the strategies used to construct and train the chosen CNN. Consequently, the reported performance varied widely, with accuracies of 91.63–100% in differentiating meningiomas, gliomas, and pituitary tumors (26 articles) and of 60.0–99.46% in distinguishing low-grade from high-grade gliomas (13 articles). The review provides a survey of the state of the art in CNN-based deep learning methods for brain tumor classification. Many networks demonstrated good performance, and it is not evident that any specific methodological choice greatly outperforms the alternatives, especially given the inconsistencies in the reporting of validation methods, performance metrics, and training data encountered. Few studies have focused on clinical usability.

## 1. Introduction

Brain tumors are a heterogenous group of common intracranial tumors that cause significant mortality and morbidity [1,2]. Malignant brain tumors are among the most aggressive and deadly neoplasms in people of all ages, with mortality rates of 5.4/100,000 men and 3.6/100,000 women per year being reported between 2014 and 2018 [3]. According to the 2021 World Health Organization (WHO) Classification of Tumors of the Central Nervous System, brain tumors are classified into four grades (I to IV) of increasingly aggressive malignancy and worsening prognosis. Indeed, in clinical practice, tumor type and grade influence treatment choice. Within WHO Grade IV tumors, glioblastoma is the most aggressive primary brain tumor, with a median survival after diagnosis of just 12–15 months [4].

The pathological assessment of tissue samples is the reference standard for tumor diagnosis and grading. However, a non-invasive tool capable of accurately classifying tumor type and of inferring grade would be highly desirable [5]. Although there are several non-invasive imaging modalities that can visualize brain tumors, i.e., Computed Tomography (CT), Positron Emission Tomography (PET), and Magnetic Resonance Imaging (MRI), the last of these remains the standard of care in clinical practice [6]. MRI conveys information on the lesion location, size, extent, features, relationship with the surrounding structures, and associated mass effect [6]. Beyond structural information, MRI can also assess microstructural features such as lesion cellularity [7], microvascular architecture [8], and perfusion [9]. Advanced imaging techniques may demonstrate many aspects of tumor heterogeneity related to type, aggressiveness, and grade; however, they are limited in assessing the mesoscopic changes that predate macroscopic ones [10]. Many molecular imaging techniques have recently been developed to better reveal and quantify heterogeneity, permitting a more accurate characterization of brain tumors. However, in order to make use of this wealth of new information, more sophisticated and potentially partially automated tools for image analysis may be useful [10].

Computer-aided detection and diagnosis (CADe and CADx, respectively), which refer to software that combines artificial intelligence and computer vision to analyze radiological and pathology images, have been developed to help radiologists diagnose human disease in several body districts, including in applications for colorectal polyp detection and segmentation [11,12] and lung cancer classification [13,14,15].

Machine learning has vigorously accelerated the development of CAD systems [16]. One of the most recent applications of machine learning in CAD is classifying objects of interest, such as lesions, into specific classes based on input features [17,18,19,20]. In machine learning, various image analysis tasks can be performed by finding or learning informative features that successfully describe the regularities or patterns in data. However, conventionally, meaningful or task-relevant features are mainly designed by human experts based on their knowledge of the target domain, making it challenging for those without domain expertise to leverage machine learning techniques. Furthermore, traditional machine learning methods can only detect superficial linear relationships, while the biology underpinning living organisms is several orders of magnitude more complex [21].

Deep learning [22], which is inspired by an understanding of the neural networks within the human brain, has achieved unprecedented success in facing the challenges mentioned above by incorporating the feature extraction and selection steps into the training process [23]. Generically, deep learning models are represented by a series of layers, and each is formed by a weighted sum of elements in the previous layer. The first layer represents the data, and the last layer represents the output or solution. Multiple layers enable complicated mapping functions to be reproduced, allowing deep learning models to solve very challenging problems while typically needing less human intervention than traditional machine learning methods. Deep learning currently outperforms alternative machine learning approaches [24] and, for the past few years, has been widely used for a variety of tasks in medical image analysis [25].

A convolutional neural network (CNN) is a deep learning approach that has frequently been applied to medical imaging problems. It overcomes the limitations of previous deep learning approaches because its architecture allows it to automatically learn the features that are important for a problem using a training corpus of sufficient variety and quality [26]. Recently, CNNs have gained popularity for brain tumor classification due to their outstanding performance with very high accuracy in a research context [27,28,29,30,31].

Despite the growing interest in CNN-based CADx within the research community, translation into daily clinical practice has yet to be achieved due to obstacles such as the lack of an adequate amount of reliable data for training algorithms and imbalances within the datasets used for multi-class classification [32,33], among others. Several reviews [31,32,33,34,35,36] have been published in this regard, summarizing the classification methods and key achievements and pointing out some of the limitations in previous studies, but as of yet, none of them have focused on the deficiencies regarding clinical adoption or have attempted to determine the future research directions required to promote the application of deep learning models in clinical practice. For these reasons, the current review considers the key limitations and obstacles regarding the clinical applicability of studies in brain tumor classification using CNN algorithms and how to translate CNN-based CADx technology into better clinical decision making.

In this review, we explore the current studies on using CNN-based deep learning techniques for brain tumor classification published between 2015 and 2022. We decided to focus on CNN architectures, as alternative deep-learning techniques, such as Deep Belief Networks or Restricted Boltzmann Machines, are much less represented in the current literature.

The objectives of the review were three-fold: to (1) review and analyze article characteristics and the impact of CNN methods applied to MRI for glioma classification, (2) explore the limitations of current research and the gaps in bench-to-bedside translation, and (3) find directions for future research in this field. This review was designed to answer the following research questions: How has deep learning been applied to process MR images for glioma classification? What level of impact have papers in this field achieved? How can the translational gap be bridged to deploy deep learning algorithms in clinical practice?

The review is organized as follows: Section 2 introduces the methods used to search and select literature related to the focus of the review. Section 3 presents the general steps of CNN-based deep learning methods for brain tumor classification, and Section 4 introduces relevant primary studies, with an overview of their datasets, preprocessing techniques, and computational methods for brain tumor classification, and presents a quantitative analysis of the covered studies. Furthermore, we introduce the factors that may directly or indirectly degrade the performance and the clinical applicability of CNN-based CADx systems and provide an overview of the included studies with reference to the degrading factors. Section 5 presents a comparison between the selected studies and suggests directions for further improvements, and finally, Section 6 summarizes the work and findings of this study.

## 2. Materials and Methods

### 2.1. Article Identification

In this review, we identified preliminary sources using two online databases, PubMed and Scopus. The search queries used to interrogate each database are described in Table 1. The filter option for the publication year (2015–2022) was selected so that only papers in the chosen period were fed into the screening process (Supplementary Materials). Searches were conducted on 30 June 2022. PubMed generated 212 results, and Scopus yielded 328 results.

### 2.2. Article Selection

Articles were selected for final review using a three-stage screening process (Supplementary Materials) based on a series of inclusion and exclusion criteria. After removing duplicate records that were generated from using two databases, articles were first screened based on the title alone. The abstract was then assessed, and finally, the full articles were checked to confirm eligibility. The entire screening process (Supplementary Materials) was conducted by one author (Y.T.X). In cases of doubt, records were reviewed by other authors (D.N.M, C.T), and the decision regarding inclusion was arrived at by consensus.

The meet the inclusion criteria, articles had to:

- Be original research articles published in a peer-reviewed journal with full-text access offered by the University of Bologna;
- Involve the use of any kind of MR images;
- Be published in English;
- Be concerned with the application of CNN deep learning techniques for brain tumor classification.

Included articles were limited to those published from 2015 to 2022 to focus on deep learning methodologies. Here, a study was defined as work that employed a CNN-based deep learning algorithm to classify brain tumors and that involved the use of one or more of the following performance metrics: accuracy, the area under the receiver operating characteristics curve, sensitivity, specificity, or F_1_ score.

Exclusion criteria were:

- Review articles;
- Book or book chapters;
- Conference papers or abstracts;
- Short communications or case reports;
- Unclear descriptions of data;
- No validation performed.

If a study involved the use of a CNN model for feature extraction but traditional machine learning techniques for the classification task, it was excluded. Studies that used other deep learning networks, for example, artificial neural networks (ANNs), generative adversarial networks (GANs), or autoencoders (AEs), instead of CNN models were excluded. Studies using multiple deep learning techniques as well as CNNs were included in this study, but only the performance of the CNNs will be reviewed.

**Figure 1:**
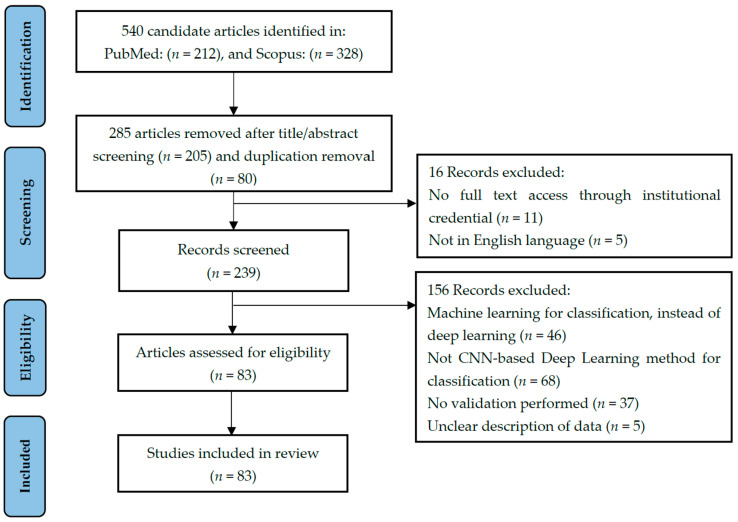
The PRISMA flowchart of this review. *n*: number of articles.

Figure 1 reports the numbers of articles screened after exclusion at each stage as per the Preferred Reporting Items for Systematic Reviews and Meta-Analyses (PRISMA) guidelines [37]. A review of 83 selected papers is presented in this paper. All of the articles cover the classification of brain tumors using CNN-based deep learning techniques.

## 3. Literature Review

This section presents a detailed overview of the research papers dealing with brain tumor classification using CNN-based deep learning techniques published during the period from 2015 to 2022. This section is formulated as follows: Section 3.1 presents a brief overview of the general methodology adopted in the majority of the papers for the classification of brain MRI images using CNN algorithms. Section 3.2 presents a description of the popular publicly available datasets that have been used in the research papers reviewed in the form of a table. Section 3.3 introduces the commonly applied preprocessing methods used in the reviewed studies. Section 3.4 provides an introduction of widely used data augmentation methods. Finally, Section 3.5 provides a brief overview of the performance metrics that provide evidence about the credibility of a specific classification algorithm model.

### 3.1. Basic Architecture of CNN-Based Methods

Recently, deep learning has shown outstanding performance in medical image analysis, especially in brain tumor classification. Deep learning networks have achieved higher accuracy than classical machine learning approaches [24]. In deep learning, CNNs have achieved significant recognition for their capacity to automatically extract deep features by adapting to small changes in the images [26]. Deep features are those that are derived from other features that are relevant to the final model output.

**Figure 2:**
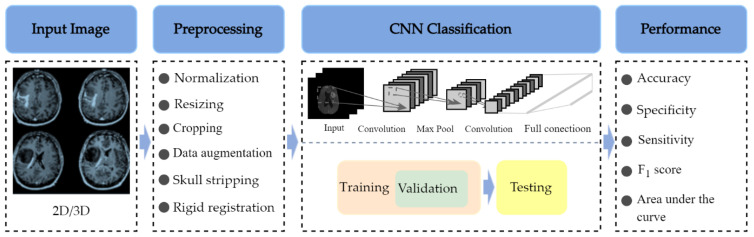
The basic workflow of a typical CNN-based brain tumor classification study with four high-level steps: Step 1. Input Image: 2D or 3D Brain MR samples are fed into the classification model; Step 2. Preprocessing: several preprocessing techniques are used to remove the skull, normalize the images, resize the images, and augment the number of training examples; Step 3. CNN Classification: the preprocessed dataset is propagated into the CNN model and is involved in training, validation, and testing processes; Step 4. Performance Evaluation: evaluation of the classification performance of a CNN algorithm with accuracy, specificity, F_1_ score, area under the curve, and sensitivity metrics.

The architecture of a typical deep CNN-based brain tumor classification frame is described in Figure 2. To train a CNN-based deep learning model with tens of thousands of parameters, a general rule of thumb is to have at least about 10 times the number of samples as parameters in the network for the effective generalization of the problem [38]. Overfitting may occur during the training process if the training dataset is not sufficiently large [39]. Therefore, many studies [40,41,42,43,44] use 2D brain image slices extracted from 3D brain MRI volumes to solve this problem, which increases the number of examples within the initial dataset and mitigates the class imbalance problem. In addition, it has the advantage of reducing the input data dimension and reducing the computational burden of training the network.

Data augmentation is another effective technique for increasing both the amount and the diversity of the training data by adding modified copies of existing data with commonly used morphological techniques, such as rotation, reflection (also referred to as flipping or mirroring), scaling, translation, and cropping [44,45]. Such strategies are based on the assumption that the size and orientation of image patches do not yield robust features for tumor classification.

In deep learning, overfitting is also a common problem that occurs when the learning capacity is so large that the network will learn spurious features instead of meaningful patterns [39]. A validation set can be used in the training process to avoid overfitting and to obtain the stable performance of the brain tumor classification system on future unseen data in clinical practice. The validation set provides an unbiased evaluation of a classification model using multiple subsets of the training dataset while tuning the model’s hyperparameters during the training process [46]. In addition, validation datasets can be used for regularization by early stopping when the error on the validation dataset increases, which is a sign of overfitting to the training data [39,47]. Therefore, in the article selection process, we excluded the articles that omitted validation during the training process.

Evaluating the classification performance of a CNN algorithm is an essential part of a research study. The accuracy, specificity, F_1_ score (also known as the Dice similarity coefficient) [48], the area under the curve, and sensitivity are important metrics to assess the classification model’s performance and to compare it to similar works in the field.

### 3.2. Datasets

A large training dataset is required to create an accurate and trustworthy deep learning-based classification system for brain tumor classification. In the current instance, this usually comprises a set of MR image volumes, and for each, a classification label is generated by a domain expert such as a neuroradiologist. In the reviewed literature, several datasets were used for brain tumor classification, targeting both binary tasks [27,40,41,45] and multiclass classification tasks [24,30,49,50,51]. Table 2 briefly lists some of the publicly accessible databases that have been used in the studies reviewed in this paper, including the MRI sequences as well as the size, classes, unbiased Gini Coefficient, and the web address of the online repository for the specific dataset.

The Gini coefficient (G) [52] is a property of distribution that measures its difference using uniformity. It can be applied to categorical data in which classes are sorted by prevalence. Its minimum value is zero if all of the classes are equally represented, and its maximum values varies between 0.5 for a two-class distribution to an asymptote of 1 for many classes. The unbiased Gini coefficient divides G by the maximum value of the number of classes present and takes values in the range of 0–1. The maximum value for a distribution with n classes is (n − 1)/n. The values of the unbiased Gini coefficient were calculated using R package DescTools [52]. Table 2 shows the characteristics of public datasets in terms of balancing the samples of the available classes of tumors (unbiased Gini coefficient) while considering the total number of samples in the datasets (“Size” column).

**Table 2:** An overview of publicly available datasets.

Dataset Name	Available Sequences	Size	Classes	Unbiased Gini Coefficient	Source
TCGA-GBM	T_1_w, ceT_1_w, T_2_w, FLAIR	199 patients	N/D	N/D	[53]
TCGA-LGG	T_1_w, ceT_1_ce, T_2_w, FLAIR	299 patients	N/D	N/D	[54]
Brain tumor dataset from Figshare (Cheng et al., 2017)	ceT_1_w	233 patients (82 MEN, 89 Glioma, 62 PT), 3064 images (708 MEN, 1426 Glioma, 930 PT)	Patients (82 MEN, 89 Glioma, 62 PT), images (708 MEN, 1426 Glioma, 930 PT)	0.116 (patients), 0.234 (images)	[55]
Kaggle (Navoneel et al., 2019)	No information given	253 images (98 normal, 155 tumorous)	98 normal, 155 tumorous	0.225	[56]
REMBRANDT	T_1_w, T_2_w, FLAIR, DWI	112 patients (30 AST-II, 17 AST-II, 14 OLI-II, 7 OLI-III, 44 GBM)	30 AST-II, 17 AST-II, 14 OLI-II, 7 OLI-III, 44 GBM	0.402	[57]
BraTS	T_1_w, ceT_1_w, T_2_w, FLAIR	2019: 335 patients (259 HGG, 76 LGG); 2018: 284 patients (209 HGG, 75 LGG); 2017: 285 patients (210 HGG, 75 LGG); 2015: 274 patients (220 HGG, 54 LGG)	2019: 259 HGG, 76 LGG;2018: 209 HGG, 75 LGG;2017: 210 HGG, 75 LGG; 2015: 220 HGG, 54 LGG	0.546 (2019); 0.472 (2018); 0.474 (2017); 0.606 (2015)	[58]
ClinicalTrials.gov (Liu et al., 2017)	T_1_w, ceT_1_w, T_2_w, FLAIR	113 patients (52 LGG, 61 HGG)	52 LGG, 61 HGG	0.080	[59]
CPM-RadPath 2019	T_1_w, ceT_1_w, T_2_w, FLAIR	329 patients	N/D	N/D	[60]
IXI dataset	T_1_w, T_2_w, DWI	600 normal images	N/D	N/D	[61]
RIDER	T_1_w, T_2_w, DCE-MRI, ce-FLAIR	19 GBM patients (70,220 images)	70,220 images	N/D	[62]
Harvard Medical School Data	T_2_w	42 patients (2 normal, 40 tumor), 540 images (27 normal, 513 tumorous)	Patients (2 normal, 40 tumorous), images (27 normal, 513 tumorous)	0.905 (patients), 0.900 (images)	[63]

Among the public datasets, the dataset from Figshare provided by Cheng [55] is the most popular dataset and has been widely used for brain tumor classification. BraTS, which refers to the Multimodal Brain Tumor Segmentation Challenge (a well-known challenge that has taken place every year since 2012), is another dataset that is often used for testing brain tumor classification methods. The provided data are pre-processed, co-registered to the same anatomical template, interpolated to the exact resolution (1 mm^3^), and skull stripped [55].

Most MR techniques can generate high-resolution images, while different imaging techniques show distinct contrast, are sensitive to specific tissues or fluid regions, and highlight relevant metabolic or biophysical properties of brain tumors [64]. The datasets listed in Table 2 collect one or more MRI sequences, including T_1_-weighted (T_1_w), T_2_-weighted (T_2_w), contrast-enhanced T_1_-weighted (ceT_1_w), fluid-attenuated inversion recovery (FLAIR), diffusion-weighted imaging (DWI), and dynamic contrast-enhanced magnetic resonance imaging (DCE-MRI) sequences. Among these, the T_1_w, T_2_w, ceT_1_w, and FLAIR sequences are widely used for brain tumor classification in both research and in clinical practice. Each sequence is distinguished by a particular series of radiofrequency pulses and magnetic field gradients, resulting in images with a characteristic appearance [64]. Table 3 lists the imaging configurations and the main clinical distinctions of T_1_w, T_2_w, ceT_1_w, and FLAIR with information retrieved from [64,65,66,67].

### 3.3. Preprocessing

Preprocessing is used mainly to remove extraneous variance from the input data and to simplify the model training task. Other steps, such as resizing, are needed to work around the limitations of neural network models.

#### 3.3.1. Normalization

The dataset fed into CNN models may be collected with different clinical protocols and various scanners from multiple institutions. The dataset may consist of MR images with different intensities because the intensities of MR image are not consistent across different MR scanners [69]. In addition, the intensity values of MR images are sensitive to the acquisition condition [70]. Therefore, input data should be normalized to minimize the influence of differences between the scanners and scanning parameters. Otherwise, any CNN network that is created will be ill-conditioned.

There are many methods for data normalization, including min-max normalization, z-score normalization, and normalization by decimal scaling [71]. Min-max normalization is one of the most common ways to normalize MR images found in the included articles [27,36,40]. In that approach, the intensity values of the input MR images are rescaled into the range of (0, 1) or (−1, 1).

Z-score normalization refers to the process of normalizing every intensity value found in MR images such that the mean of all of the values is 0 and the standard deviation is 1 [71].

#### 3.3.2. Skull Stripping

MRI images of the brain also normally contain non-brain regions such as the dura mater, skull, meninges, and scalp. Including these parts in the model typically deteriorates its performance during classification tasks. Therefore, in the studies on brain MRI datasets that retain regions of the skull and vertebral column, skull stripping is widely applied as a preprocessing step in brain tumor classification problems to improve performance [24,72,73].

#### 3.3.3. Resizing

Since deep neural networks require inputs of a fixed size, all of the images need to be resized before being fed into CNN classification models [74]. Images larger than the required size can be downsized by either cropping the background pixels or by downscaling using interpolation [74,75].

#### 3.3.4. Image Registration

Image registration is defined as a process that spatially transforms different images into one coordinate system. In brain tumor classification, it is often necessary to analyze multiple images of a patient to improve the treatment plan, but the images may be acquired from different scanners, at different times, and from different viewpoints [76]. Registration is necessary to be able to integrate the data obtained from these different measurements.

Rigid image registration is one of the most widely utilized registration methods in the reviewed studies [77,78]. Rigid registration means that the distance between any two points in an MR image remains unchanged before and after transformation. This approach only allows translation and rotation transformations. 

#### 3.3.5. Bias Field Correction

In medical images, the bias field is an undesirable artifact caused by factors such as the scan position and instrument used as well as by other unknown issues [79]. This artifact is characterized by differences in brightness across the image and can significantly degrade the performance of many medical image analysis techniques. Therefore, a preprocessing step is needed to correct the bias field signal before submitting corrupted MR images to a CNN classification model.

The N4 bias field correction algorithm and the Statistical Parametric Mapping (SPM) module are common approaches for correcting the inhomogeneity in the intensity of MR images. The N4 bias field correction algorithm is a popular method for correcting the low-frequency-intensity non-uniformity present in MR image data [80]. SPM contains several software packages that are used for brain segmentation. These packages usually contain a set for skull stripping, intensity non-uniformity (bias) correction, and segmentation routines [81].

### 3.4. Data Augmentation

**Figure 3:**
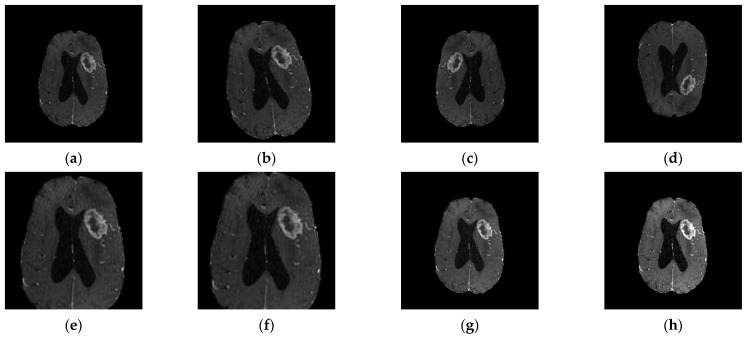
Data augmentation: (**a**) original image; (**b**) 18° rotation. When rotating by an arbitrary number of degrees (non-modulo 90), rotation will result in the image being padded in each corner. Then, a crop is taken from the center of the newly rotated image to retain the largest crop possible while maintaining the image’s aspect ratio; (**c**) left–right flipping; (**d**) top–bottom flipping; (**e**) scaling by 1.5 times; (**f**) cropping by center cropping to the size 150 × 150; (**g**) random brightness enhancement; (**h**) random contrast enhancement.

CNN-based classification requires a large number of data. A general rule of thumb is to have at least about 10 times the number of samples set as parameters in the network for the effective generalization of the problem [38]. If the database is significantly smaller, overfitting might occur. Data augmentation is one of the foremost data techniques to subside imbalanced distribution and data scarcity problems. It has been used in many studies focusing brain tumor classification [24,45,49,50] and involves geometrical transformation operations such as rotation, reflection (also referred to as flipping or mirroring), scaling, translation, and cropping (Figure 3).

Data augmentation techniques can be divided into two classes: position augmentation and color augmentation. Some of the most popular position augmentation methods include rotation, reflection (also referred to as flipping or mirroring), scaling, translation, and cropping, and they have been commonly used to enlarge MR datasets in studies focusing on brain tumor classification [45,51,72,77]. Color augmentation methods such as contrast enhancement and brightness enhancement have also been applied in the included studies [28,43].

Recently, well-established data augmentation techniques have begun to be supplemented by automatic methods that use deep learning approaches. For example, the authors in [44] proposed a progressively growing generative adversarial network (PGGAN) augmentation model to help overcome the shortage of images needed for CNN classification models. However, such methods are rare in the literature reviewed.

### 3.5. Performance Measures

Evaluating the classification performance of a CNN algorithm is an essential part of a research study. Here, we outline the evaluation metrics that are the most commonly encountered in the brain tumor classification literature, namely accuracy, precision, sensitivity, F1 score, and the area under the curve.

In classification tasks, true positive (*TP*) represents an image that is correctly classified into the positive class according to the ground truth. Similarly, true negative is an outcome in which the model correctly classifies an imagine into the negative class. On the other hand, false positive (*FP*) is an outcome in which the model incorrectly classifies an image into the positive class when the ground truth is negative. False negative (*FN*) is an outcome in which the model incorrectly classifies an image that should be placed in the positive class.

#### 3.5.1. Accuracy

Accuracy (*ACC*) is a metric that measures the performance of a model in correctly classifying the classes in a given dataset and is given as the percentage of total correct classifications divided by the total number of images.

(1) $$ACC=\frac{TP+TN}{TP+TN+FP+FN}$$

#### 3.5.2. Specificity

Specificity (*SPE*) represents the proportion of correctly classified negative samples to all of the negative samples identified in the data.

(2) $$SPE=\frac{TN}{TN+FP}$$

#### 3.5.3. Precision

Precision (*PRE*) represents the ratio of true positives to all of the identified positives.

(3) $$PRE=\frac{TP}{TP+FP}$$

#### 3.5.4. Sensitivity

Sensitivity (*SEN*) measures the ability of a classification model to identify positive samples. It represents the ratio of true positives to the total number of (actual) positives in the data.

(4) $$SEN=\frac{TP}{TP+FN}$$

#### 3.5.5. F_1_ Score

The *F*_1_ score [48] is one of the most popular metrics and considers both precision and recall. It can be used to assess the performance of classification models with class imbalance problems [82] and considers the number of prediction errors that a model makes and looks at the type of errors that are made. It is higher if there is a balance between *PRE* and *SEN*.

(5) $$F_{1}\;\;\mathrm{score}=2\frac{PRE\times SEN}{PRE+SEN}$$

#### 3.5.6. Area under the Curve

The area under the curve (AUC) measures the entire two-dimensional area underneath the ROC curve from (0, 0) to (1, 1). It measures the ability of a classifier to distinguish between classes.

Clinicians and software developers need to understand how performance metrics can measure the properties of CNN models for different medical problems. In research studies, several metrics are typically used to evaluate a model’s performance.

Accuracy is among the most commonly used metric to evaluate a classification model but is also known for being misleading in cases when the classes have different distributions in the data [83,84]. Precision is an important metric in cases when the occurrence of false positives is unacceptable/intolerable [84]. Specificity measures the ability of a model to correctly identify people without the disease in question. Sensitivity, also known as recall, is an important metric in cases where identifying the number of positives is crucial and when the occurrence of false negatives is unacceptable/intolerable [83,84]. It must be interpreted with care in cases with strongly imbalanced classes.

It is important to recognize that there is always a tradeoff between sensitivity and specificity. Balancing between two metrics has to be based on the medical use case and the associated requirements [83]. Precision and sensitivity are both proportional to TP but have an inverse relationship. Whether to maximize recall or precision depends on the application: Is it more important to only identify relevant instances, or to make sure that all relevant instances are identified? The balance between precision and sensitivity has to be considered in medical use cases in which some false positives are tolerable; for example, in cancer detection, it is crucial to identify all positive cases. On the other hand, for a less severe disease with high prevalence, it is important to achieve the highest possible precision [83].

## 4. Results

This section provides an overview of the research papers focusing on brain tumor classification using CNN techniques. Section 4.1 presents a quantitative analysis of the number of articles published from 2015 to 2022 on deep learning and CNN in brain tumor classification and the usage of the different CNN algorithms applied in the studies covered. Then, Section 4.2 introduces the factors that may directly or indirectly degrade the performance and the clinical applicability of CNN-based CADx systems. Finally, in Section 4.3, an overview of the included studies will be provided with reference to the degrading factors introduced in Section 4.2.

### 4.1. Quantitative Analysis

**Figure 4:**
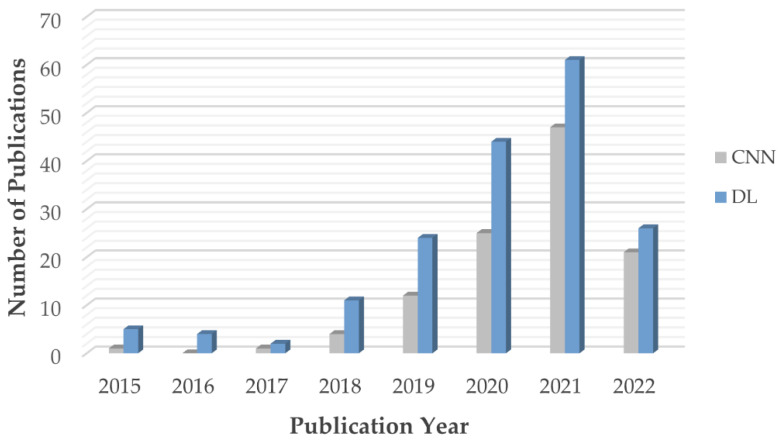
Number of articles published from 2015 to 2022.

As mentioned in the introduction, many CNN models have been used to classify the MR images of brain tumor patients. They overcome the limitations of earlier deep learning approaches and have gained popularity among researchers for brain tumor classification tasks. Figure 4 shows the number of research articles on brain tumor classification using deep learning methods and CNN-based deep learning techniques published on PubMed and Scopus in the years from 2015 to June 2022; the number of papers related to brain tumor classification using CNN techniques grows rapidly from 2019 onwards and accounts for the majority of the total number of studies published in 2020, 2021, and 2022. This is because of the high generalizability, stability, and accuracy rate of CNN algorithms.

**Figure 5:**
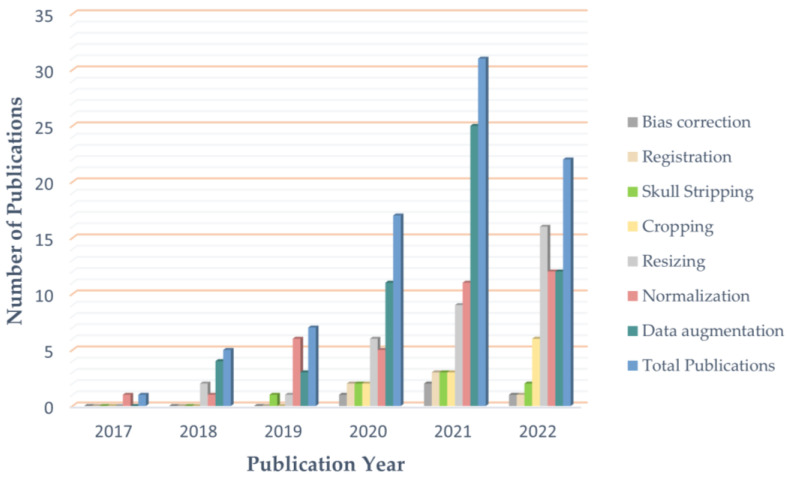
Usage of preprocessing techniques from 2017 to 2022.

Figure 5 shows the usage of the most commonly used preprocessing techniques for addressing problems in brain tumor classification, including data augmentation, normalization, resizing, skull stripping, bias field correction, and registration. In this figure, only data from 2017 to 2022 are visualized, as no articles using the preprocessing methods mentioned were published in 2015 or 2016. Since 2020, data augmentation has been used in the majority of studies to ease data scarcity and overfitting problems. However, the bias field problem has yet to be taken seriously, and few studies have included bias field correction in the preprocessing process.

**Figure 6:**
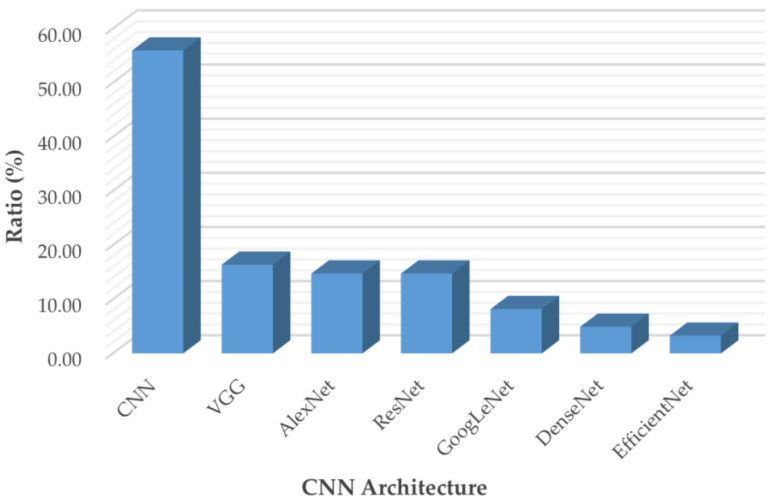
Usage of state-of-the-art CNN models from 2015 and 2022.

Figure 6 breaks down the usage of the publicly available CNN architectures used in the articles included in this review, including custom CNN models, VGG, AlexNet, ResNet, GoogLeNet, DenseNet, and EfficientNet.

AlexNet [85] came out in 2012 and was a revolutionary advancement in deep learning; it improved traditional CNNs by introducing a composition of consecutively stacked convolutional layers and became one of the best models for image classification. VGG, which refers to the Visual Geometry Group, was a breakthrough in the world of convolutional neural networks after AlexNet. It is a type of deep CNN architecture with multiple layers that was originally proposed by K. Simonyan and A. Zisserman in [86] and was developed to improve model performance by increasing the depth of such CNNs.

GoogLeNet is a deep convolutional neural network with 22 layers based on the Inception architecture; it was developed by researchers at Google [87]. GoogLeNet addresses most of the problems that large networks face, such as computational expense and overfitting, by employing the Inception module. This module can use max pooling and three varied sizes of filters (1 × 1, 3 × 3, 5 × 5) for convolution in a single image block; such blocks are then concatenated and passed onto the next layer. An extra 1 × 1 convolution can be added to the neural network before the 3 × 3 and 5 × 5 layers to make the process even less computationally expensive [87]. ResNet stands for Deep Residual Network. It is an innovative convolutional neural network that was originally proposed in [88]. ResNet makes use of residual blocks to improve the accuracy of models. A residual block is a skip-connection block that typically has double- or triple-layer skips that contain nonlinearities (ReLU) and batch normalization in between; it can help to reduce the problem of vanishing gradients or can help to mitigate accuracy saturation problems [88]. DenseNet, which stands for Dense Convolutional Network, is a type of convolutional neural network that utilizes dense connections between layers. DenseNet was mainly developed to improve the decreased accuracy caused by the vanishing gradient in neural networks [89]. Additionally, those CNNs take in images with a pixel resolution of 224 × 224. Therefore, for brain tumor classification, the authors need to center crop a 224 × 224 patch in each image to keep the input image size consistent.

Convolutional neural networks are commonly built using a fixed resource budget. When more resources are available, the depth, width, and resolution of the model need to be scaled up for better accuracy and efficiency [90]. Unlike previous CNNs, EfficientNet is a novel baseline network that uses a different model-scaling technique based on a compound coefficient and neural architecture search methods that can carefully balance network depth, width, and resolution [90].

### 4.2. Clinical Applicability Degrading Factors

This section introduces the factors that hinder the adoption and development of CNN-based brain tumor classification CADx systems into clinic practice, including data quality, data scarcity, data mismatch, data imbalance, classification performance, research value towards clinic needs, and the Black-Box characteristics of CNN models.

#### 4.2.1. Data Quality

During the MR image acquisition process, both the scanner and external sources may produce electrical noise in the receiver coil, generating image artifacts in the brain MR volumes [69]. In addition, the MR image reconstruction process is sensitive to acquisition conditions, and further artifacts are introduced if the subject under examination moves during the acquisition of a single image [69]. These errors are inevitable and reduce the quality of the MR images used to train networks. As a result, the quality of the training data degrades the sensitivity/specificity of CNN models, thus compromising their applicability in a clinic setting.

#### 4.2.2. Data Scarcity

Big data is one of the biggest challenges that CNN-based CADx systems face today. A large number of high-quality annotated data is required to build high-performance CNN classification models, while it is a challenge to label a large number of medical images due to the complexity of medical data. When a CNN classification system does not have enough data, overfitting can occur—as classification is based on extraneous variance in the training set—affecting the capacity of the network to generalize new data [91].

#### 4.2.3. Data Mismatch

Data mismatch refers to a situation in which a model that has been well-trained in a lab environment fails to generalize real-world clinical data. It might be caused by overfitting of the training set or due to mismatch between research images and clinic ones [82]. Studies are at high risk of generalization failure if they omit a validation step or if the test set does not reflect the characteristics of the clinical data.

#### 4.2.4. Class Imbalance

In brain MRI datasets such as the BraTS 2019 dataset [92], which consists of 210 HGG and 75 LGG patients (unbiased Gini coefficient 0.546, as shown in Table 2), HGG is represented by a much higher percentage of samples than LGG, leading to so-called class imbalance problems, in which inputting all of the data into the CNN classifier to build up the learning model will usually lead to a learning bias to the majority class [93]. When an unbalanced training set is used, it is important to assess model performance using several performance measures (Section 3.5).

#### 4.2.5. Research Value towards Clinical Needs

Different brain tumor classification tasks were studied using CNN-based deep learning techniques during the period from 2015 to 2022, including clinically relevant two-class classification (normal vs. tumorous [29,41,94,95], HGG vs. LGG [27,40,45,73], LGG-II vs. LGG-III [96], etc.); three-class classification (normal vs. LGG vs. HGG [24], meningioma (MEN) vs. pituitary tumor (PT) vs. glioma [39,42,49,50], glioblastoma multiforme (GBM) vs. astrocytoma (AST) vs. oligodendroglioma (OLI) [30], etc.); four-class classification (LGG vs. OLI vs. anaplastic glioma (AG) vs. GBM [72], normal vs. AST-II vs. OLI-III vs. GBM-IV [24], normal vs. MEN vs. PT vs. glioma [97], etc.); five-class classification (AST-II vs. AST-III vs. OLI-II vs. OLI-III vs. GBM-IV [24]); and six-class classification (normal vs. AST-II vs. AST-III vs. OLI-II vs. OLI-III vs. GBM-IV [24]).

Not all classification tasks are equally difficult, and this is the case for the deep learning research community and clinical practice. The authors in [24] used AlexNet for multi-class classification tasks, including two-class classification: normal vs. tumor, three-class classification: normal vs. LGG vs. HGG; four-class classification: normal vs. AST vs. OLI vs. GBM; five-class classification: AST-II vs. AST-III vs. OLI-II vs. OLI-III vs. GBM-IV, and six-class classification: normal vs. AST-II vs. AST-III vs. OLI-II vs. OLI-III vs. GBM-IV. The results reported 100% accuracy for the normal vs. tumorous classification. The accuracy for the five-class classification (AST-II vs. AST-III vs. OLI-II vs. OLI-III vs. GBM-IV) was only 87.14%. Similarly, in a recent publication [98], the authors utilized the same CNN model for multi-class brain tumor classification. The overall accuracy obtained for normal vs. tumorous classification reached 100% compared to the lower accuracy of 90.35% obtained for the four-class classification task (Grade I vs. Grade II vs. Grade III vs. Grade IV) and 86.08% for the five-class classification of AST-II vs. AST-III vs. OLI-II vs. OLI-III vs. GBM.

The goal of research in the field of CADx is to help address existing unmet clinical needs and to provide assistance methods and tools for the difficult tasks that human professionals cannot easily handle in clinical practice. It is observed that CNN-based models have achieved quite high accuracies for normal/tumorous image classification, while more research is needed to improve the classification performance of more difficult tasks, especially in five-class classification (e.g., AST-II vs. AST-III vs. OLI-II vs. OLI-III vs. GBM) and four-class classification (e.g., Grade I vs. Grade II vs. Grade III vs. Grade IV) tasks. Therefore, studies that use normal vs. tumorous as their target problem have little clinical value.

#### 4.2.6. Classification Performance

Classification performance, which indicates the reliability and trustworthiness of CADx systems, is one of the most important factors to be considered when translating research findings into clinical practice. It has been shown that CNN techniques perform well in most of brain tumor classification tasks, such as in two-class classification (normal and tumorous [94,95] and HGG and LGG [45,73]) and three-class classification (normal vs. LGG vs. HGG [24] and MEN vs. PT vs. glioma [49,50]) tasks. However, the classification performance obtained for more difficult classification tasks, such as a five-class classification between AST-II, AST-III, OLI-II, OLI-III, and GBM, remains poor [24,98] and justifies further research.

#### 4.2.7. Black-Box Characteristics of CNN Models

The brain tumor classification performance of some of the CNN-based deep learning techniques reviewed here is remarkable. Still, their clinical application is also limited by another factor: the “Black-Box” problem. Even the designers of a CNN model cannot usually explain the internal workings of the model or why it arrived at a specific decision. The features used to decide the classification of any given image are not an output of the system. This lack of explainability reduces the confidence of clinicians in the results of the techniques and impedes the adoption and development of deep learning tools into clinical practice [99].

### 4.3. Overview of Included Studies

Many research papers have emerged following the wave of enthusiasm for CNN-based deep learning techniques from 2015 to present day. In this review, 83 research papers are assessed to summarize the effectiveness of CNN algorithms in brain tumor classification and to suggest directions for future research in this field.

Among the articles included, twenty-five use normal/tumorous as their classification target. However, as mentioned in Section 4.2.5, the differentiation between normal and tumorous images is not a difficult task. It has been well-solved both in research and clinic practice and thus has little value for clinical application. Therefore, studies that use normal vs. tumorous as their target problem will not be reviewed further in the following assessment steps.

Table 4a provides an overview of the included studies that focus on CNN-based deep learning methods for brain tumor classification but does not include studies working with a normal vs. tumorous classification. The datasets, MRI sequences, size of the datasets, and the preprocessing methods are summarized. Table 4b summarizes the classification tasks, classification architecture, validation methods, and performance metrics of the reviewed articles.

As introduced in Section 4.2, the major challenge confronting brain tumor classification using CNN techniques in MR images lies in the training data, including the challenges caused by data quality, data scarcity, data mismatch, and data imbalance, which hinder the adoption and development of CNN-based brain tumor classification CADx systems into clinic practice. Here, we assess several recently published studies to provide a convenient collection of the state-of-the-art techniques that have been used to address these issues and the problems that have not been solved in those studies.

Currently, data augmentation is recognized as the best solution to the problem caused by data scarcity and has been widely utilized in brain tumor classification studies.

The authors in [100] used different data augmentation methods, including rotation, flipping, Gaussian blur, sharpening, edge detection, embossing, skewing, and shearing, to increase the size of the dataset. The proposed system aims to classify between Grade I, Grade II, Grade III, and Grade IV, and the original data consist of 121 images (36 Grade I images, 32 Grade II images, 25 Grade III images, and 28 Grade IV images), and by using data augmentation techniques, 30 new images are generated from each MR image. The proposed model is experimentally evaluated using both augmented and original data. The results show that the overall accuracy after data augmentation reaches 90.67%, which is greater than the accuracy of 87.38% obtained without augmentation.

While most data augmentation techniques aim to increase extraneous variance in the training set, deep learning can be used by itself, at least in theory, to increase meaningful variance. In a recent publication by Allah et al. [44], a novel data augmentation method called a progressive growing generative adversarial network (PGGAN) was proposed and combined with rotation and flipping methods. The method involves an incremental increase of the size of the model during the training to produce MR images of brain tumors and to help overcome the shortage of images for deep learning training. The brain tumor images were classified using a VGG19 feature extractor coupled with a CNN classifier. The accuracy of the combined VGG19 + CNN and PGGAN data augmentation framework achieved an accuracy of 98.54%.

Another approach that helps overcome the problem of data scarcity and that can also reduce computational costs and training time is transfer learning. Transfer learning is a hot research topic in machine learning; previously learned knowledge can be transferred for the performance of a new task by fine-tuning a previously generated model with a smaller dataset that is more specific to the aim of the study. Transfer learning is usually expressed using pre-trained models such as VGG, GoogLeNet, and AlexNet that have been trained on the large benchmark dataset ImageNet [101].

**Table 4:** (a) Overview of included studies that focus on CNN-based deep learning methods for brain tumor classification, with the exception of studies focusing on normal vs. tumorous classification. Datasets, MRI sequences, size of the datasets, and preprocessing methods are summarized. (b) Overview of included studies that focus on CNN-based deep learning methods for brain tumor classification, with the exception of study focusing on normal vs. tumorous classification. Classification tasks, classification architecture, validation methods, and performance metrics are summarized.

(a)																	
Author and Year	Datasets	MRI Sequences	Size of Dataset		Pre-Processing						Data Augmentation						
			Patients	Images	Cropping	Normalization	Resizing	Skull Stripping	Registration ^1^	Other	Translation ^2^	Rotation	Scaling ^3^	Reflection ^4^	Shearing	Cropping	Other (X = Unspecified)
Özcan et al. [27] 2021	Private dataset	T_2_w/FLAIR	104 (50 LGG, 54 HGG)	518	x	x				Conversion to BMP		x	x	x	x		
Hao et al. [102] 2021	BraTS 2019	T_1_w, ceT_1_w, T_2_w	335 (259 HGG, 76 LGG)	6700			x	x	x								
Tripathi et al. [103] 2021	1. TCGA-GBM, 2. LGG-1p19qDeletion	T_2_w	322 (163 HGG, 159 LGG)	7392 (5088 LGG, 2304 HGG)				x			x	x	x	x		x	
Ge et al. [40] 2020	BraTS 2017	T_1_w, ceT_1_w, T_2_w, FLAIR	285 (210 HGG, 75 LGG)								x			x			
Mzoughi et al. [28] 2020	BraTS 2018	ceT_1_w	284 (209 HGG, 75 LGG)			x	x			Contrast enhancement				x			
Yang et al. [45] 2018	ClinicalTrials.gov (NCT026226201)	ceT_1_w	113 (52 LGG, 61 HGG)							Conversion to BMP		x	x	x			Histogram equalization, adding noise
Zhuge et al. [77] 2020	1.TCIA-LGG, 2. BraTS 2018	T_1_w, T_2_w, FLAIR, ceT_1_w	315 (210 HGG, 105 LGG)			x			x	Clipping, bias field correction		x	x	x			
Decuyper et al. [73] 2021	1. TCGA-LGG, 2. TCGA-GBM, 3. TCGA-1p19qDeletion, 4. BraTS 2019. 5. GUH dataset	T_1_w, ceT_1_w, T_2_w, FLAIR	738 (164 from TCGA-GBM, 121 from TCGA-LGG, 141 from 1p19qDeletion, 202 from BraTS 2019, 110 from GUH dataset) (398 GBM vs. 340 LGG)			x		x	x	Interpolation		x		x			Elastic transform
He et al. [78] 2021	1.Dataset from TCIA	FLAIR, ceT_1_w	214 (106 HGG, 108 LGG)			x	x		x								x
	2. BraTS 2017	FLAIR, ceT_1_w	285 (210 HGG, 75 LGG)			x	x		x								x
Hamdaoui et al. [104] 2021	BraTS 2019	T_1_w, ceT_1_w, T_2_w, FLAIR	285 (210 HGG, 75 LGG)	53,064 (26,532 HGG, 26,532 LGG)	x								x	x			
Chikhalikar et al. [105] 2021	BraTS 2015	T_2_w, FLAIR	274 (220 HGG, 54 LGG)	521						Contrast enhancement							
Ahmad [106] 2019	BraTS 2015	No info shared		124 (99 HGG, 25 LGG)		x											
Naser et al. [96] 2020	TCGA-LGG	T_1_W, FLAIR, ceT_1_w	108 (50 Grade II, 58 Grade III)		x	x	x			Padding	x	x	x	x	x		
Allah et al. [44] 2021	Figshare (Cheng et al., 2017)	ceT_1_w	233 (as shown in Table 2)	3064 (as shown in Table 2)		x						x		x			PGGAN
Swati et al. [50] 2019	Figshare (Cheng et al., 2017)	ceT_1_w	233 (as shown in Table 2)	3064 (as shown in Table 2)		x	x										
Guan et al. [43] 2021	Figshare (Cheng et al., 2017)	ceT_1_w	233 (as shown in Table 2)	3064 (as shown in Table 2)		x	x			Contrast enhancement		x		x			
Deepak et al. [39] 2019	Figshare (Cheng et al., 2017)	ceT_1_w	233 (as shown in Table 2)	3064 (as shown in Table 2)		x	x										
Díaz-Pernas et al. [42] 2021	Figshare (Cheng et al., 2017)	ceT_1_w	233 (as shown in Table 2)	3064 (as shown in Table 2)		x											Elastic transform
Ismael et al. [49] 2020	Figshare (Cheng et al., 2017)	ceT_1_w	233 (as shown in Table 2)	3064 (as shown in Table 2)	x		x				x	x	x	x	x		Whitening, brightness manipulation
Alhassan et al. [107] 2021	Figshare (Cheng et al., 2017)	ceT_1_w	233 (as shown in Table 2)	3064 (as shown in Table 2)		x											
Bulla et al. [108] 2020	Figshare (Cheng et al., 2017)	ceT_1_w	233 (as shown in Table 2)	3064 (as shown in Table 2)		x	x										
Ghassemi et al. [109] 2020	Figshare (Cheng et al., 2017)	ceT_1_w	233 (as shown in Table 2)	3064 (as shown in Table 2)		x						x		x			
Kakarla et al. [110] 2021	Figshare (Cheng et al., 2017)	ceT_1_w	233 (as shown in Table 2)	3064 (as shown in Table 2)		x	x			Contrast enhancement							
Noreen et al. [111] 2021	Figshare (Cheng et al., 2017)	ceT_1_w	233 (as shown in Table 2)	3064 (as shown in Table 2)		x											
Noreen et al. [112] 2020	Figshare (Cheng et al., 2017)	ceT_1_w	233 (as shown in Table 2)	3064 (as shown in Table 2)		x											
Kumar et al. [113] 2021	Figshare (Cheng et al., 2017)	ceT_1_w	233 (as shown in Table 2)	3064 (as shown in Table 2)								x					
Badža et al. [114] 2020	Figshare (Cheng et al., 2017)	ceT_1_w	233 (as shown in Table 2)	3064 (as shown in Table 2)		x	x					x		x			
Alaraimi et al. [115] 2021	Figshare (Cheng et al., 2017)	ceT_1_w	233 (as shown in Table 2)	3064 (as shown in Table 2)		x	x				x	x	x	x		x	
Lo et al. [116] 2019	Dataset from TCIA **	ceT_1_w	130 (30 Grade II, 43 Grade III, 57 Grade IV)			x	x			Contrast enhancement	x	x	x	x		x	
Kurc et al. [117] 2020	Data from TCGA	ceT_1_w, T_2_-FLAIR	32 (16 OLI, 16 AST)					x	x	Bias field correction		x				x	
Pei et al. [118] 2020	1. CPM-RadPath 2019, 2. BraTS 2019	T_1_w, ceT_1_w, T_2_w, FLAIR	398 (329 from CPM-RadPath 2019, 69 from BraTS 2019)			x		x	x	Noise reduction		x	x			x	
Ahammed et al. [72] 2019	Private dataset	T_2_w	20	557 (130 Grade I, 169 Grade II, Grade III 103, Grade IV 155)				x		Filtering, enhancement	x	x	x	x			
Mohammed et al. [51] 2020	Radiopaedia	No info shared	60 (15 of each class)	1258 (311 EP, 286 normal, 380 MEN, 281 MB)			x			Denoising	x	x	x	x		x	
McAvoy et al. [119] 2021	Private dataset	ceT_1_w	320 (160 GBM, 160 PCNSL)	3887 (2332 GBM, 1555 PCNSL)		x	x			Random changes to color, noise sampling				x			
Gilanie et al. [120] 2021	Private dataset	T_1_w, T_2_w, FLAIR	180 (50 AST-I, 40 AST-II, 40 AST-III, 50 AST-IV)	30240 (8400 AST-I, 6720 AST-II, 6720 AST-III, 8400 AST-IV)		x				Bias field correction		x					
Kulkarni et al. [121] 2021	Private dataset	T_1_w, T_2_w, FLAIR		200 (100 benign, 100 malignant)						Denoising, contrast enhancement	x	x	x	x	x		
Artzi et al. [122] 2021	Private dataset	T_1_w, FLAIR, DTI	158 (22 Normal, 63 PA, 57 MB, 16 EP)	731 (110 Normal, 280 PA, 266 MB, 75 EP)	x		x		x	Background removal, bias field correction		x	x	x			Brightness changes
Tariciotti et al. [123] 2022	Private dataset	ceT1w	121 (47 GBM, 37 PCNSL, 37 Metastasis)	3597 (1481 GBM, 1073 PCNSL, 1043 Metastasis))		x	x			Conversion to PNG							
Ait et al. [124] 2022	Figshare (Cheng et al., 2017)	ceT_1_w	233 (as shown in Table 2)	3064 (as shown in Table 2)		x	x										
Alanazi et al. [125] 2022	1. Dataset from Kaggle	No info shared		826 Glioma, 822 MEN, 395 no tumor, and 827 PT	x	x	x			Noise removal							
	2. Figshare (Cheng et al., 2017)	ceT_1_w	233 (as shown in Table 2)	3064 (as shown in Table 2)	x	x	x			Noise removal							
Ye et al. [126] 2022	Private dataset	ceT_1_w	73			x	x			Image transformation				x			Blurring, ghosting, motion, affining, random elastic deformation
Gaur et al. [127] 2022	MRI dataset by Bhuvaji	No info shared		2296			x			Gaussian noise adding							
Guo et al. [128] 2022	CPM-RadPath 2020	T_1_w, ceT_1_w, T_2_w, FLAIR	221 (133 GBM, 54 AST, 34 OLI)					x	x	Bias field correction, Gaussian noise adding		x	x				Random contrast adjusting
Aamir et al. [129] 2022	Figshare (Cheng et al., 2017)	ceT_1_w	233 (as shown in Table 2)	3064 (as shown in Table 2)		x				Contrast enhancement		x		x			
Rizwan et al. [130] 2022	Figshare (Cheng et al., 2017)	ceT_1_w	230 (81 MEN, 90 Glioma, 59 PT)	3061 (707 MEN, 1425 Glioma, 929 PT)	x		x			Noise filtering and smoothing							salt-noise/grayscale di stortion
	Dataset from TCIA	T_1_w	513 (204 Grade II, 128 Grade III, 181 Grade IV)	70 (32 Grade II, 18 Grade III, 20 Grade IV)	x		x			Noise filtering and smoothing							salt-noise/grayscale di stortion
Nayak et al. [131] 2022	1.daataset from Kaggle, 2. Figshare (Cheng et al., 2017)	ceT_1_w	1. No info shared, 2. 233 (as shown in Table 2)	3260 (196 Normal, 3064 (as shown in Table 2))		x				Gaussian blurring, noise removal	x	x	x				
Chatterjee et al. [132] 2022	1.BraTS2019, 2. IXI Dataset	ceT_1_w	1. 332 (259 HGG, 73 LGG), 2. 259 Normal			x	x	x						x			Affine
Khazaee et al. [133] 2022	BraTS2019	ceT_1_w, T_2_w, FLAIR	335 (259 HGG, 76 LGG)	26,904 (13,233 HGG, 13,671 LGG)								x		x			
Isunuri et al. [134] 2022	Figshare (Cheng et al., 2017)	ceT_1_w	233 (as shown in Table 2)	3064 (as shown in Table 2)		x	x										
Gu et al. [30] 2021	1. Figshare (Cheng et al., 2017)	ceT_1_w	233 (as shown in Table 2)	3064 (as shown in Table 2)			x										
	2. REMBRANDT	No info shared	130	110,020			x										
Rajini [135] 2019	1. IXI dataset, REMBRANDT, TCGA-GBM, TCGA-LGG	No info shared	600 normal images from IXI dataset, 130 patients from REMBRANDT, 200 patients from TCGA-GBM, 299 patients from TCGA-LGG														
	2. Figshare (Cheng et al., 2017)	ceT_1_w	233 (as shown in Table 2)	3064 (as shown in Table 2)													
Anaraki et al. [136] 2019	1: IXI dataset, REMBRANDT, TCGA-GBM, TCGA-LGG, private dataset	no info of IXI, ceT_1_w from REMBRANDT, TCGA-GBM, TCGA-LGG	600 normal images from IXI dataset, 130 patients from REMBRANDT, 199 patients from TCGA-GBM, 299 patients from TCGA-LGG, 60 patients from private dataset			x	x				x	x	x	x			
	2. Figshare (Cheng et al., 2017)	ceT_1_w	233 (as shown in Table 2)	3064 (as shown in Table 2)		x	x				x	x	x	x			
Sajjad et al. [100] 2019	1. Radiopaedia	No info shared		121 (36 Grade I, 32 Grade II, 25 Grade III, 28 Grade IV)		x	x			Denoising, bias field correction		x		x	x		Gaussian blurring, sharpening, embossing, skewing
	2. Figshare (Cheng et al., 2017)	ceT_1_w	233 (as shown in Table 2)	3064 (as shown in Table 2)		x	x			Denoising, bias field correction		x		x	x		Gaussian blurring, sharpening, embossing, skewing
Wahlang et al. [137] 2020	1. Radiopaedia	FLAIR	11 (2 Metastasis, 6 Glioma, 3 MEN)													x	
	2. BraTS 2017	No info shared	20	3100						Median filtering							
Tandel et al. [138] 2021	REMBRANDT	T_2_w	See 1–4 below	See 1–4 below			x			Converted to RGB		x	x				
			130	1. 2156 (1041 normal, 1091 tumorous)													
			47	2. 557 (356 AST-II, 201 AST-III)													
			21	3. 219 (128 OLI-II, 91 OLI-III)													
			112	4. 1115 (484 LGG, 631 HGG)													
Xiao et al. [97] 2021	1. Private dataset	No info shared		1109 (495 MT, 614 Normal)			x										
	2. Figshare (Cheng et al., 2017)	ceT_1_w	233 (as shown in Table 2)	3064 (as shown in Table 2)			x										
	3. Brain Tumor Classification (MRI) Dataset from Kaggle	No info shared		3264 (937 MEN, 926 Glioma, 901 PT, 500 Normal)			x										
Tandel et al. [24] 2020	REMBRANDT	T_2_w	112 (30 AST-II, 17 AST-II, 14 OLI-II, 7 OLI-III, 44 GBM)	See 1–5 below				x				x	x				
				1. 2132 (1041 normal, 1091 tumorous)													
				2. 2156 (1041 normal, 484 LGG, 631 HGG)													
				3. 2156 (1041 normal, 557 AST, 219 OLI, 339 GBM)													
				4. 1115 (356 AST-II, 201 AST-III, 128 OLI-II, 91 OLI-III, 339 GBM) 5. 2156 (1041 normal, 356 AST-II, 201 AST-III, 128 OLI-II, 91 OLI-III, 339 GBM)													
			
Ayadi et al. [98] 2021	1. Radiopaedia	No info shared		121 (36 Grade I, 32 Grade II, 25 Grade III, 28 Grade IV)								x		x			Gaussian blurring, sharpening
	2. Figshare (Cheng et al., 2017)	ceT_1_w	233 (as shown in Table 2)	3064 (as shown in Table 2)													
	3. REMBRANDT	FLAIR, T_1_w, T_2_w	130 (47 AST, 21 OLI, 44 GBM, 18 unknown)	See 1–5 below								x		x			Gaussian blurring, sharpening
				1. 2132 (1041 normal, 1091 tumorous) 2. 2156 (1041 normal, 484 LGG, 631 HGG) 3. 2156 (1041 normal, 557 AST, 219 OLI, 339 GBM) 4. 1115 (356 AST-II, 201 AST-III, 128 OLI-II, 91 OLI-III, 339 GBM) 5. 2156 (1041 normal, 356 AST-II, 201 AST-III, 128 OLI-II, 91 OLI-III, 339 GBM)													
			
			
			
			
**(b)**																	
**Author and Year**		**Classification Tasks**		**Model Architecture**		**Validation**				**Performance**							**ACC% ^5^**
*2 classes*																	
Özcan et al. [27] 2021		LGG (grade II) vs. HGG (grade IV)		Custom CNN model		5-fold CV				SEN = 98.0%, SPE = 96.3%, F1 score = 97.0%, AUC = 0.989							97.1
Hao et al. [102] 2021		LGG vs. HGG		Transfer learning with AlexNet		No info shared				AUC = 82.89%							
Tripathi et al. [103] 2021		LGG vs. HGG		Transfer learning with Resnet18		No info shared											95.87
Ge et al. [40] 2020		LGG vs. HGG		Custom CNN model		No info shared				SEN = 84.35%, SPE = 93.65%							90.7
Mzoughi et al. [28] 2020		LGG vs. HGG		Multi-scale 3D CNN		No info shared											96.49
Yang et al. [45] 2018		LGG vs. HGG		Transfer learning with AlexNet, GoogLeNet		5-fold CV				AUC = 0.939							86.7
Zhuge et al. [77] 2020		LGG vs. HGG		Transfer learning with ResNet50		5-fold CV				SEN = 93.5%, SPE = 97.2%							96.3
				3D CNN		5-fold CV				SEN = 94.7%, SPE = 96.8%							97.1
Decuyper et al. [73] 2021		LGG vs. GBM		3D CNN		No info shared				SEN = 90.16%, SPE = 89.80%, AUC = 0.9398							90
He et al. [78] 2021		LGG vs. HGG		Custom CNN model		5-fold CV				TCIA: SEN = 97.14%, SPE = 90.48%, AUC = 0.9349							92.86
										BraTS 2017: SEN = 95.24%, SPE = 92%, AUC = 0.952							94.39
Hamdaoui et al. [104] 2021		LGG vs. HGG		Transfer learning with stacking VGG16, VGG19, MobileNet, InceptionV3, Xception, Inception ResNetV2, DenseNet121		10-fold CV				PRE = 98.67%, F1 score = 98.62%, SEN = 98.33%							98.06
Chikhalikar et al. [105] 2021		LGG vs. HGG		Custom CNN model		No info shared											99.46
Ahmad [106] 2019		LGG vs. HGG		Custom CNN model		No info shared											88
Khazaee et al. [133] 2022		LGG vs. HGG		Transfer learning with EfficientNetB0		CV				PRE = 98.98%, SEN = 98.86%, SPE = 98.79%							98.87%
Naser et al. [96] 2020		LGG (Grade II) vs. LGG (Grade III)		Transfer learning with VGG16		5-fold CV				SEN = 97%, SPE = 98%							95
Kurc et al. [117] 2020	OLI vs. AST		3D CNN		5-fold CV						80						
McAvoy et al. [119] 2021		GBM vs. PCNSL		Transfer learning with EfficientNetB4		No info shared				GBM: AUC = 0.94, PCNSL: AUC = 0.95							
Kulkarni et al. [121] 2021		Benign vs. Malignant		Transfer learning with AlexNet		5-fold CV				PRE = 93.7%, RE = 100%, F1 score = 96.77%							96.55
				Transfer learning with VGG16		5-fold CV				PRE = 55%, RE = 50%, F1 score = 52.38%							50
				Transfer learning with ResNet18		5-fold CV				PRE = 78.94%, RE = 83.33%, F1 score = 81.07%							82.5
				Transfer learning with ResNet50		5-fold CV				PRE = 95%, RE = 55.88%, F1 score = 70.36%							60
				Transfer learning with GoogLeNet		5-fold CV				PRE = 75%, RE = 100%, F1 score = 85.71%							87.5
Wahlang et al. [137] 2020		HGG vs. LGG		AlexNet		No info shared											62
				U-Net		No info shared											60
Xiao et al. [97] 2021		MT vs. Normal		Transfer learning with ResNet50		3-fold, 5-fold, 10-fold CV				AUC = 0.9530							98.2
Alanazi et al. [125] 2022		Normal vs. Tumorous		Custom CNN		No info shared											95.75%
Tandel et al. [138] 2021		1. Normal vs. Tumorous		DL-MajVot (AlexNet, VGG16, ResNet18, GoogleNet, ResNet50)		5-fold CV				SEN = 96.76%, SPE = 96.43%, AUC = 0.966							96.51
		2. AST-II vs. AST-III		DL-MajVot (AlexNet, VGG16, ResNet18, GoogleNet, ResNet50)		5-fold CV				SEN = 94.63%, SPE = 99.44%, AUC = 0.9704							97.7
		3. OLI-II vs. OLI-III		DL-MajVot (AlexNet, VGG16, ResNet18, GoogleNet, ResNet50)		5-fold CV				SEN = 100%, SPE = 100%, AUC = 1							100
		4. LGG vs. HGG		DL-MajVot (AlexNet, VGG16, ResNet18, GoogleNet, ResNet50)		5-fold CV				SEN = 98.33%, SPE = 98.57%, AUC = 0.9845							98.43
Tandel et al. [24] 2020		Normal vs. Tumorous		Transfer learning with AlexNet		Multiple CV (K2, K5, K10)				RE = 100%, PRE = 100%, F1 score = 100%							100
Ayadi et al. [98] 2021		Normal vs. Tumorous		Custom CNN model		5-fold CV											100
Ye et al. [126] 2022		Germinoma vs. Glioma		Transfer learning with ResNet18		5-fold CV				AUC = 0.88							81%
3 classes																	
Allah et al. [44] 2021		MEN vs. Glioma vs. PT		PGGAN-augmentation VGG19		No info shared											98.54
Swati et al. [50] 2019		MEN vs. Glioma vs. PT		Transfer learning with VGG19		5-fold CV				SEN = 94.25%, SPE = 94.69%, PRE = 89.52%, F1 score = 91.73%							94.82
Guan et al. [43] 2021		MEN vs. Glioma vs. PT		EfficientNet		5-fold CV											98.04
Deepak et al. [39] 2019		MEN vs. Glioma vs. PT		Transfer learning with GoogleNet		5-fold CV											98
Díaz-Pernas et al. [42] 2021		MEN vs. Glioma vs. PT		Multiscale CNN		5-fold CV											97.3
Ismael et al. [49] 2020		MEN vs. Glioma vs. PT		Residual networks		5-fold CV				PRE = 99.0%, RE = 99.0%, F1 score = 99.0%							99
Alhassan et al. [107] 2021		MEN vs. Glioma vs. PT		Custom CNN model		k-fold CV				PRE = 99.6%, RE = 98.6%, F1 score = 99.0%							98.6
Bulla et al. [108] 2020		MEN vs. Glioma vs. PT		Transfer learning with InceptionV3 CNN model		holdout validation, 10-fold CV, stratified 10-fold CV, group 10-fold CV				Under group 10-fold CV: PRE = 97.57%, RE = 99.47%, F1 score = 98.40%, AUC = 0.995							99.82
Ghassemi et al. [109] 2020		MEN vs. Glioma vs. PT		CNN-GAN		5-fold CV				PRE = 95.29%, SEN = 94.91%, SPE = 97.69%, F1 score = 95.10%							95.6
Kakarla et al. [110] 2021		MEN vs. Glioma vs. PT		Custom CNN model		5-fold CV				PRE = 97.41%, RE = 97.42%							97.42
Noreen et al. [111] 2021		MEN vs. Glioma vs. PT		Transfer learning with Inception-v3		K-fold CV											93.31
				Transfer learning with Inception model		K-fold CV											91.63
Noreen et al. [112] 2020		MEN vs. Glioma vs. PT		Transfer learning with Inception-v3		No info shared											99.34
				Transfer learning with DensNet201		No info shared											99.51
Kumar et al. [113] 2021		MEN vs. Glioma vs. PT		Transfer learning with ResNet50		5-fold CV				PRE = 97.20%, RE = 97.20%, F1 score = 97.20%							
Badža et al. [114] 2020		MEN vs. Glioma vs. PT		Custom CNN model		10-fold CV				PRE = 95.79%, RE = 96.51%, F1 score = 96.11%							96.56
Ait et al. [124] 2022		MEN vs. Glioma vs. PT		Custom CNN		No info shared				PRE = 98.3%, SEN = 98.6%, F1 score = 98.6%							98.70%
Alanazi et al. [125] 2022		MEN vs. Glioma vs. PT		Custom CNN		No info shared											96.90%
Gaur et al. [127] 2022		MEN vs. Glioma vs. PT		Custom CNN		k-fold CV											94.64%
Aamir et al. [129] 2022		MEN vs. Glioma vs. PT		Custom CNN		5-fold CV											98.95%
Rizwan et al. [130] 2022		MEN vs. Glioma vs. PT		Custom CNN		No info shared											99.8%
Isunuri et al. [134] 2022		MEN vs. Glioma vs. PT		Custom CNN		5-fold CV				PRE = 97.33%, SEN = 97.19%, F1 score = 97.26%							97.52%
Alaraimi et al. [115] 2021		MEN vs. Glioma vs. PT		Transfer learning with AlexNet		No info shared				AUC = 0.976							94.4
				Transfer learning with VGG16		No info shared				AUC = 0.981							100
				Transfer learning with GoogLeNet		No info shared				AUC = 0.986							98.5
Lo et al. [116] 2019		Grade II vs. Grade III vs. Grade IV		Transfer learning with AlexNet		10-fold CV											97.9
Pei et al. [118] 2020		GBM vs. AST vs. OLI		3D CNN		No info shared											74.9
Gu et al. [30] 2021		1. MEN vs. Glioma vs. PT		Custom CNN model		5-fold CV				SEN = 94.64%, PRE = 94.61%, F1 score = 94.70%							96.39
		2. GBM vs. AST vs. OLI		Custom CNN model		5-fold CV				SEN = 93.66%, PRE = 95.12%, F1 score = 94.05%							97.37
Rajini [135] 2019		MEN vs. Glioma vs. PT		Custom CNN model		5-fold CV											98.16
Anaraki et al. [136] 2019		MEN vs. Glioma vs. PT		Custom CNN model		5-fold CV											94.2
Sajjad et al. [100] 2019		MEN vs. Glioma vs. PT		Transfer learning with VGG19		No info shared				SEN = 88.41%, SPE = 96.12%							94.58
Wahlang et al. [137] 2020		Metastasis vs. Glioma vs. MEN		Lenet		No info shared											48
				AlexNet		No info shared											75
Xiao et al. [97] 2021		MEN vs. Glioma vs. PT		Transfer learning with ResNet50		3-fold, 5-fold, 10-fold CV											98.02
Tandel et al. [24] 2020		Normal vs. LGG vs. HGG		Transfer learning with AlexNet		Multiple CV (K2, K5, K10)				RE = 94.85%, PRE = 94.75%, F1 score = 94.8%							95.97
Chatterjee et al. [132] 2022		Normal vs. HGG vs. LGG		Transfer learning with ResNet		3-fold CV				F1 score = 93.45%							96.84%
Ayadi et al. [98] 2021		1. Normal vs. LGG vs. HGG		Custom CNN model		5-fold CV											95
		2. MEN vs. Glioma vs. PT		Custom CNN model		5-fold CV											94.74
Guo et al. [128] 2022		GBM vs. AST vs. OLI		Custom CNN		3-fold CV				SEN = 0.772, SPE = 93.0%, AUC = 0.902							87.8%
Rizwan et al. [130] 2022		Grade I vs. Grade II vs. Grade III		Custom CNN		No info shared											97.14%
Tariciotti et al. [123] 2022		Metastasis vs. GBM vs. PCNSL		Resnet101		Hold-out				PRE = 91.88%, SEN = 90.84%, SPE = 96.34%, F1 score = 91.0%, AUC = 0.92							94.72%
4 classes																	
Ahammed et al. [72] 2019		Grade I vs. Grade II vs. Grade III vs. Grade IV		VGG19		No info shared				PRE = 94.71%, SEN = 92.72%, SPE = 98.13%, F1 score = 93.71%							98.25
Mohammed et al. [51] 2020		EP vs. MEN vs. MB vs. Normal		Custom CNN model		No info shared				SEN = 96%, PRE = 100%							96
Gilanie et al. [120] 2021		AST-I vs. AST-II vs. AST-III vs. AST-IV		Custom CNN model		No info shared											96.56
Artzi et al. [122] 2021		Normal vs. PA vs. MB vs. EP		Custom CNN model		5-fold CV											88
Nayak et al. [131] 2022		Normal vs. MEN vs. Glioma vs. PT		Transfer learning with EfficientNet		No info shared				PRE = 98.75%, F1 score = 98.75%							98.78%
Rajini [135] 2019		Normal vs. Grade II vs. Grade III vs. Grade IV		Custom CNN model		5-fold CV											96.77
Anaraki et al. [136] 2019		Normal vs. Grade II vs. Grade III vs. Grade IV		Custom CNN model		5-fold CV											
Sajjad et al. [100] 2019		Grade I vs. Grade II vs. Grade III vs. Grade IV		Transfer learning with VGG19		No info shared											90.67
Xiao et al. [97] 2021		MEN vs. Glioma vs. PT vs. Normal		Transfer learning with ResNet50		3-fold, 5-fold, 10-fold CV				PRE = 97.43%, RE = 97.67%, SPE = 99.24%, F1 score = 97.55%							97.7
Tandel et al. [24] 2020		Normal vs. AST vs. OLI vs. GBM		Transfer learning with AlexNet		Multiple CV (K2, K5, K10)				RE = 94.17%, PRE = 95.41%, F1 score = 94.78%							96.56
Ayadi et al. [98] 2021		1. normal vs. AST vs. OLI vs. GBM		Custom CNN model		5-fold CV											94.41
		2. Grade I vs. Grade II vs. Grade III vs. Grade IV		Custom CNN model		5-fold CV											93.71
5 classes																	
Tandel et al. [24] 2020		AST-II vs. AST-III vs. OLI-II vs. OLI-III vs. GBM-IV		Transfer learning with AlexNet		Multiple CV (K2, K5, K10)				RE = 84.4%, PRE = 89.57%, F1 score = 86.89%							87.14
Ayadi et al. [98] 2021		AST-II vs. AST-III vs. OLI-II vs. OLI-III vs. GBM		Custom CNN model		5-fold CV											86.08
6 classes																	
Tandel et al. [24] 2020		Normal vs. AST-II vs. AST-III vs. OLI-II vs. OLI-III vs. GBM-IV		Transfer learning with AlexNet		Multiple CV (K2, K5, K10)				RE = 91.51%, PRE = 92.46%, F1 score = 91.97%							93.74
Ayadi et al. [98] 2021		normal vs. AST-II vs. AST-III vs. OLI-II vs. OLI-III vs. GBM		Custom CNN model		5-fold CV											92.09

Notes: ^1^ Rigid registration unless otherwise notes; ^2^ translation also referred to as shifting; ^3^ scaling also referred to as zooming; ^4^ reflection also referred to as flipping or mirroring; ** The Cancer Imaging Archive, https://www.cancerimagingarchive.net/ (accessed on 27 July 2022). ^5^ Referring to overall accuracy, mean accuracy, or highest accuracy depending on the information provided by the paper or the highest accuracy when multiple models are used.

Many attempts have been made to investigate the value of transfer learning techniques for brain tumor classification [39,45,50,102,104,108,116,121]. Deepak and Ameer [39] used the GoogLeNet with the transfer learning technique to differentiate between glioma, MEN, and PT from the dataset provided by Cheng [55]. This proposed system achieved a mean classification accuracy of 98%.

In a study conducted by Yang et al. [45], AlexNet and GoogLeNet were both trained from scratch and fine-tuned from pre-trained models from the ImageNet database for HGG and LGG classification. The dataset used in this method consisted of ceT_1_w images from 113 patients (52 LGG, 61 HGG) with pathologically proven gliomas. The results show that GoogLeNet proved superior to AlexNet for the task. The performance measures, including validation accuracy, test accuracy, and test AUC of GoogLeNet trained from scratch, were 0.867, 0.909, and 0.939, respectively. With fine-tuning, the pre-trained GoogLeNet obtained performed better during glioma grading, with a validation accuracy of 0.867, a test accuracy of 0.945, and a test AUC 0.968.

The authors in [50] proposed a block-wise fine-tuning strategy using a pre-trained VGG19 for brain tumor classification. The dataset consisted of 3064 images (708 MEN, 1426 glioma, and 930 PT) from 233 patients (82 MEN, 89 glioma, and 62 PT). The authors achieved an overall accuracy of 94.82% under five-fold cross-validation. In another study by Bulla et al. [108], classification was performed in a pre-trained InceptionV3 CNN model using data from the same dataset. Several validation methods, including holdout validation, 10-fold cross-validation, stratified 10-fold cross-validation, and group 10-fold cross-validation, were used during the training process. The best classification accuracy of 99.82% for patient-level classification was obtained under group 10-fold cross-validation.

The authors in [104] used InceptionResNetV2, DenseNet121, MobileNet, InceptionV3, Xception, VGG16, and VGG19, which have already been pre-trained on the ImageNet dataset, to classify HGG and LGG brain images. The MR images used in this research were collected from the BraTS 2019 database, which contains 285 patients (210 HGG, 75 LGG). The 3D MRI volumes from the dataset were then converted into 2D slices, generating 26,532 LGG images and 94,284 HGG images. The authors selected 26,532 images from HGG to balance these two classes to reduce the impact on classification performance due to class imbalance. The average precision, f1-score, and sensitivity for the test dataset were 98.67%, 98.62%, and 98.33%, respectively.

Lo et al. [116] used transfer learning with fine-tuned AlexNet and data augmentation to classify Grade II, Grade III, and Grade IV brain tumor images from a small dataset comprising 130 patients (30 Grade II, 43 Grade III, 57 Grade IV). The results demonstrate much higher accuracy when using the pre-trained AlexNet. The proposed transferred DCNN CADx system achieved a mean accuracy of 97.9% and a mean AUC of 0.9991, while the DCNN without pre-trained features only achieved a mean accuracy of 61.42% and a mean AUC of 0.8222.

Kulkarni and Sundari [121] utilized five transfer learning architectures, AlexNet, VGG16, ResNet18, ResNet50, and GoogLeNet, to classify benign and malignant brain tumors from the private dataset collected by the authors, which only contained 200 images (100 benign and 100 malignant). In addition, data augmentation techniques, including scaling, translation, rotation, translation, shearing, and reflection, were performed to generalize the model and to reduce the possibility of overfitting. The results show that the fine-tuned AlexNet architecture achieved the highest accuracy and sensitivity values of 93.7% and 100%.

Despite many studies on CADx systems demonstrating inspiring classification performance, the validation of their algorithms for clinical practice has hardly been carried out. External validation is an efficient approach to overcome the problems caused by data mismatch and to improve the generalization, stability, and robustness of classification algorithms. It is the action of evaluating the classification model in a new independent dataset to determine whether the model performs well. However, we only found two studies that used an external clinical dataset to evaluate the effectiveness and generalization capability of the proposed scheme, which is described in below.

Decuyper et al. [73] proposed a 3D CNN model to classify brain MR volumes collected from the TCGA-LGG, TCGA-GBM, and BraTS 2019 databases into HGG and LGG. Multiple MRI sequences, including T_1_w, ceT_1_w, T_2_w, and FLAIR, were used in this research. All of the MR data were co-registered to the same anatomical template and interpolated to 1 mm^3^ voxel sizes. Additionally, a completely independent dataset of 110 patients acquired at the Ghent University Hospital (GUH) was used as an external dataset to validate the efficiency and generalization of the proposed model. The resulting validation accuracy, sensitivity, specificity, and AUC for the GUH dataset were 90.00%, 90.16%, 89.80%, and 0.9398.

In [120], Gilanie et al. presented an automatic method using a CNN architecture for astrocytoma grading between AST-I, AST-II, AST-III, and AST-IV. The dataset consisted of MR slices from 180 subjects, including 50 AST-I cases, 40 AST-II cases, 40 AST-III cases, and 50 AST-IV cases. T1w, T2w, and FLAIR were used in the experiments. In addition, the N4ITK method [80] was used in the preprocessing stage to correct the bias field distortion present in the MR images. The results were validated on a locally developed dataset to evaluate the effectiveness and generalization capabilities of the proposed scheme. The proposed method obtained an overall accuracy of 96.56% for the external validation dataset.

In brain tumor classification, it is often necessary to use image co-registration to preprocess input data when images are collected from different sequences or different scanners. However, we found that this problem has not yet been taken seriously. In the surveyed articles, six studies [73,76,98,118,135,136] used data from multiple datasets for one classification target, while only two studies [73,76] performed image co-registration during the image preprocessing process.

The authors in [76] proposed a 2D Mask RCNN model and a 3DConvNet model to distinguish between LGG (Grades II and Grade III) and HGG (Grade IV) on multiple MR sequences, including T_1_w, ceT_1_w, T_2_w, and FLAIR. The TCIA-LGG and BraTS 2018 databases were used to train and validate these two CNN models in this research work. In the 2D Mask RCNN model, all of the input MR images were first preprocessed by rigid image registration and intensity inhomogeneity correction. In addition, data augmentation was also implemented to increase the size and the diversity of the training data. The performance measures accuracy, sensitivity, and specificity achieved values of 96.3%, 93.5%, and 97.2% using the proposed 2D Mask RCNN-based method and 97.1%, 94.7%, and 96.8% with the 3DConvNet method, respectively.

In the study conducted by Ayadi [98], the researchers built a custom CNN model for multiple classification tasks. They collected data from three online databases, Radiopaedia, the dataset provided by Cheng, and REMBRANDT, for brain tumor classification, but no image co-registration was performed to minimize shift between images and to reduce its impact on the classification performance. The overall accuracy obtained for tumorous and normal classification reached 100%; for normal, LGG, and HGG classification, it reached 95%; for MEN, glioma, and PT classification, it reached 94.74%; for normal, AST, OLI, and GBM classification, it reached 94.41%; for Grade I, Grade II, Grade III, and Grade IV classification, it reached 90.35%; for AST-II, AST-III, OLI-II, OLI-III, and GBM classification, it reached 86.08%; and for normal, AST-II, AST-III, OLI-II, OLI-III, and GBM classification, it reached 92.09%.

The authors in [118] proposed a 3D CNN model for brain tumor classification between GBM, AST, and OLI. A merged dataset comprising data from the CPM-RadPath 2019 and BraTS 2019 databases was used to train and validate the proposed model, but the authors did not perform image co-registration. The results show that the classification model has very poor performance during brain tumor classification, with an accuracy of 74.9%.

In [135], the researchers presented a CNN-PSO method for two classification tasks: normal vs. Grade II vs. Grade III vs. Grade IV and MEN vs. glioma vs. PA. The MR images used for the first task were collected from four publicly available datasets: the IXI dataset, REMBRANDT, TCGA-GBM, and TCGA-LGG. The overall accuracy obtained was 96.77% for classification between normal, Grade II, Grade III, and Grade IV and 98.16% for MEN, glioma, and PA classification.

Similar to the work conducted in [135], Anaraki et al. [136] used MR data merged from four online databases: the IXI dataset, REMBRANDT, TCGA-GBM, and TCGA-LGG, and from one private dataset collected by the authors for normal, Grade II, Grade III, and Grade IV classification. They also used the dataset proposed by Cheng [55] for MEN, glioma, and PA classification. Different data augmentation methods were performed to further enlarge the size of the training set. The authors in these studies did not co-register the MR images from different sequences from different institutions for the four-class classification task. The results show that 93.1% accuracy was achieved for normal, Grade II, Grade III, and Grade IV classification, and 94.2% accuracy was achieved for MEN, glioma, and PA classification.

Despite the high accuracy levels reported in most studies using CNN techniques, we found that in several studies [102,117,118,137], the models demonstrated very poor performance during brain tumor classification tasks.

The authors in [102] explored transfer learning techniques for brain tumor classification. The experiments were performed on the BraTS 2019 dataset, which consists of 335 patients diagnosed with brain tumors (259 patients with HGG and 76 patients with LGG). The model achieved a classification AUC of 82.89% on a separate test dataset of 66 patients. The classification performance obtained by transfer learning in this study is relatively low, hindering its development and application in clinical practice. The authors of [117] presented a 3D CNN model developed to categorize adult diffuse glioma cases into the OLI and AST classes. The dataset used in the experiment consisted of 32 patients (16 patients with OLI and 16 patients with AST). The model achieved accuracy values of 80%. The main reason for the poor performance probably lies in the small dataset, with only 32 patients being used for model training. That is far from enough to train a 3D model.

In another study [137], two brain tumor classification tasks were studied using the Lenet, AlexNet, and U-net CNN architectures. In the experiments, MR images from 11 patients (two metastasis, six glioma, and three MEN) obtained from Radiopaedia were utilized to classify metastasis, glioma, and MEN; the data of 20 patients collected from BraTS 2017 were used for HGG and LGG classification. The results show poor classification performance by the three CNN architectures on the two tasks, with an accuracy of 75% obtained by AlexNet and an accuracy of 48% obtained by Lenet for the first task and an accuracy of 62% obtained by AlexNet and an accuracy of 60% obtained by U-net for the second task. The poor performance of Lenet is probably due to its simple architecture, which is not capable of high-resolution image classification. On the other hand, the U-net CNN performs well in segmentation tasks but is not the most commonly used network for classification.

Even though CNNs have demonstrated remarkable performance in brain tumor classification tasks in the majority of the reviewed studies, their level of trustworthiness and transparency must be evaluated in a clinic context. Of the included articles, only two studies, conducted by Artzi et al. [122] and Gaur et al. [127], investigated the Black-Box nature of CNN models for brain tumor classification to ensure that the model is looking in the correct place rather than at noise or unrelated artifacts.

The authors in [122] proposed a pre-trained ResNet-50 CNN architecture to classify three posterior fossa tumors from a private dataset and explained the classification decision by using gradient-weighted class activation mapping (Grad-CAM). The dataset consisted of 158 MRI scans of 22 healthy controls and 63 PA, 57 MB, and 16 EP patients. In this study, several preprocessing methods were used to reduce the influence of MRI data on the classification performance of the proposed CNN model. Image co-registration was performed to ensure that the images become spatially aligned. Bias field correction was also conducted to remove the intensity gradient from the image. Data augmentation methods, including flipping, reflection, rotation, and zooming, were used to increase the size and diversity of the dataset. However, class imbalance within the dataset, particularly the under-representation of EP, was not addressed. The proposed architecture achieved a mean validation accuracy of 88% and 87% for the test dataset. The results demonstrate that the proposed network using Grad-CAM can identify the area of interest and train the classification model based on pathology-related features.

Gaur et al. [127] proposed a CNN-based model integrated with local interpretable model-agnostic explanation (LIME) and Shapley additive explanation (SHAP) for the classification and explanation of meningioma, glioma, pituitary, and normal images using an MRI dataset of 2870 MR images. For better classification results, Gaussian noise was introduced in the pre-processing step to improve the learning for the CNN, with mean = 0 and a standard deviation of 10 ^0.5^. The proposed CNN architecture achieved an accuracy of 94.64% for the MRI dataset. The proposed model also provided a locally model-agnostic explanation to describe the results for ordinary people more qualitatively. 

## 5. Discussion

**Figure 7:**
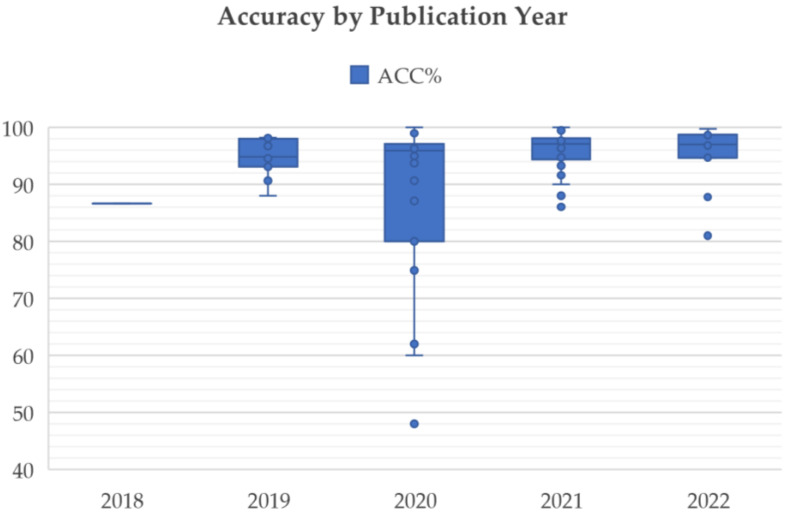
Classification accuracy by publication year.

Many of the articles included in this review demonstrate that CNN-based architectures can be powerful and effective when applied to different brain tumor classification tasks. Table 4b shows that the classification of HGG and LGG images and the differentiation of MEN, glioma, and PT images were the most frequently studied applications. The popularity of these applications is likely linked to the availability of well-known and easily accessible public databases, such as the BraTS datasets and the dataset made available by Cheng [55]. Figure 7 reveals that there is an increase in the overall accuracy achieved by CNN architectures for brain tumor classification from 2018 to 2022. It is observed that from 2019 onwards, the overall classification accuracy achieved in most studies reached 90%, with only few works obtaining lower accuracies, and in 2020, the extreme outlier accuracy was 48% [137]. It is also apparent from this figure that the proportion of papers with an accuracy higher than 95% increases after 2020.

In order to discuss the technical differences and points of similarity between the papers included in the present review, we decided to proceed thematically. Wherever possible, it is more useful to make comparisons between studies containing as few differences as possible. The most commonly reported metric, and the only one that will be employed here, is the accuracy. There are several studies that allow us to make such comparisons across only one factor. In other cases, several studies employ a similar methodology, and we can perform across-study comparisons. Finally, accuracy data can be plotted for single factors to allow for a simple visual comparison without attempting to separate confounding factors.

### 5.1. The Importance of the Classification Task

**Figure 8:**
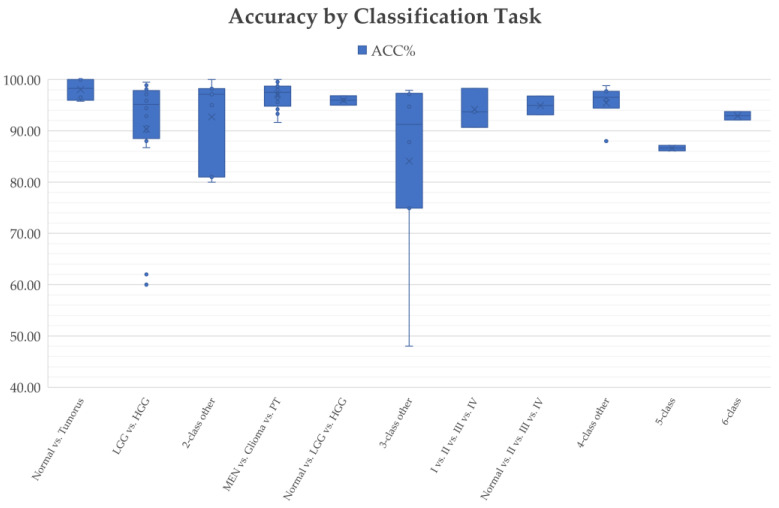
Classification accuracy by classification task.

Three papers [24,97,98] investigated the effect of splitting a dataset into different numbers of categories. They all showed the expected monotonic decrease in accuracy as the number of classes increased, with the caveat that the “normal” image category is relatively easy to distinguish from the others and does not decrease accuracy when added as an additional category. The pattern is also apparent in Figure 8—the maximum accuracy for two-class problems was 100%; for four-class problems, it was 98.8%; and for six-class problems, it was 93.7%.

Two papers employed a single architecture to perform different classification tasks [30,138] while keeping the number of classes constant. The results in [30] showed little difference between the accuracy obtained for two different problems, which could be explained by differences in the datasets. The results of [138] showed slightly larger variation between four two-class problems. Curiously, nets trained on larger datasets yielded worse accuracy values, suggesting that results obtained from smaller samples have an inflated accuracy (100% for a problem based on 219 images, 96.1% for a problem based on 2156 images). With reference to Figure 8, the classification task seems to have a larger effect than the class number on the accuracy. Note that the categories that group various specific tasks (two-class, three-class) together show much greater heterogeneity than those with the same number of classes for specific comparisons.

Further evidence regarding the importance of the task comes from a comparison of the accuracy in the papers comparing tumor grade (LGC vs. HGC) and those seeking to differentiate different types of tumors (MEN vs. glioma vs. PT); although the latter task involves more classes, the median accuracy is 97.6 (against 94.4 for the former). We compared the articles that studied the classification of HGG and LGG and found that the classification performance varies widely, even between the articles published in 2021 that utilized state-of-the-art CNN techniques. One of the key factors that significantly affects the performance of CNN models for brain tumor classification lies in the size of the datasets. The authors of [40,78] both proposed custom CNN models to classify HGG and LGG images of 285 MRI scans from the BraTS 2017 dataset. The overall accuracy values were 90.7% and 94.28%, respectively. The authors of [137] utilized AlexNet for the same task, but MRI data of only 20 patients from the same dataset were studied. The model in this study yielded a poor classification accuracy of 62%, the lowest value among the articles on this classification task.

Figure 8 presents the overall accuracies achieved by the reviewed studies that worked on different classification tasks. What stands out in the figure is that with the exception of the five-class tasks, which achieved accuracies lower than 90%, the CNNs achieved promising accuracies on different brain tumor classification tasks, especially in three-class classification tasks distinguishing between MEN, glioma, and PT. We also noticed that the accuracies of the three-class classification tasks fluctuated widely, with the lowest accuracy being 48% in [137] for the metastasis vs. glioma vs. MEN classification. More research attention should be paid to improving the accuracies of these classification tasks.

### 5.2. The Effect of the Dataset

A few studies applied the same network architecture to two different datasets. For He et al. [78], the results demonstrating a higher accuracy (94.4% against 92.9%) were based on a training set that was both larger and more unbalanced. The first factor would have improved the training process, while the latter made the classification task easier. Several papers derive different subgroups from different datasets (for example, healthy subject data from IXI and tumors from other sets). This is poor practice, as there are likely to be non-pathological differences between the sets acquired from different centres, and this can artificially inflate classification accuracy [139].

As was mentioned in the Results section, dataset size is considered a critical factor in determining the classification performance of a CNN architecture. Some studies report the dataset size in terms of the number of subjects included, and others report it in terms of the number of images. Typically, several images are included from each subject, but this number is not specified.

**Figure 9:**
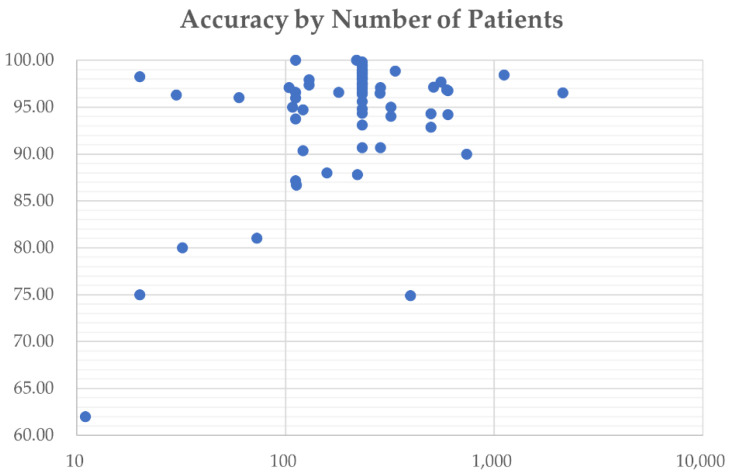
Classification accuracy by number of patients.

**Figure 10:**
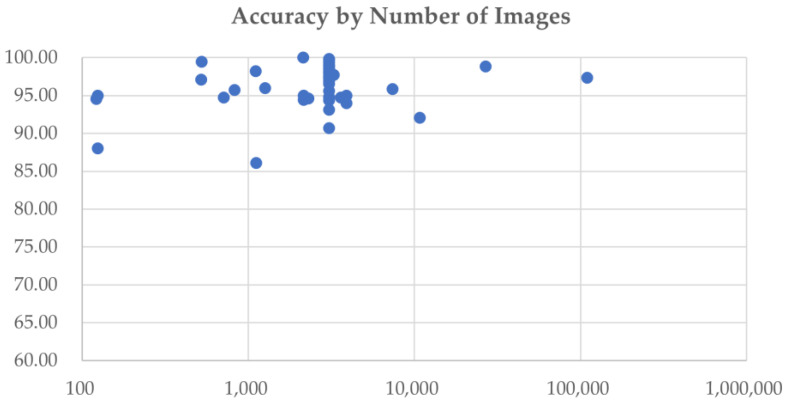
Classification accuracy by number of images.

Figure 9 and Figure 10 sum up the classification accuracies obtained according to each of the factors; Figure 9 shows that there is a marked increase in the overall accuracy achieved with more training subjects The improvement gained by increasing the image number seems more modest.

Another interesting aspect of the datasets used is the choice of MRI sequence. This may provide a hint as to the features being used for classification. Comparing the articles that focused on the same classification task, of the sequences listed in Table 3, only ceT_1_w was associated with studies showing a higher classification accuracy than those that excluded it for MEN vs. Glioma vs. PT classification, while all of the sequences contributed to an improvement in LGG vs. HGG classification. As a consequence, studies using multiple sequences were associated with higher accuracy in the LGG vs. HGG task but not in MEN vs. Glioma vs. PT classification.

### 5.3. The Effect of CNN Architecture

Three studies present comparisons of different architectures trained on the same problems (Yang et al. [45], Kulkarni et al. [121], Wahling et al. [137]).

In a study conducted by Yang et al. [45], GoogLeNet and AlexNet were both trained from scratch and fine-tuned from pre-trained models from the ImageNet database for HGG and LGG classification. When both were trained from scratch, GoogLeNet proved superior to AlexNet for the task. The test accuracies were 0.909 and 0.855, respectively. Fine-tuning pre-existing nets resulted in better performance in both cases, with accuracies on the test set of 0.945 and 0.927, respectively. In [121], five nets were used to distinguish benign from malignant tumors. The reported accuracies were surprisingly variable; from worst to best, the results were VGG16 (0.5) and ResNet50 (0.68). In [137], AlexNet and LeNet were both used to distinguish three classes.

**Figure 11:**
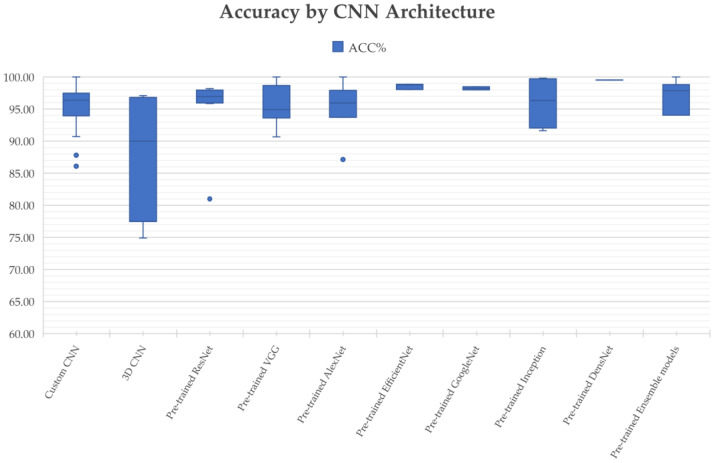
Classification accuracy by CNN architecture.

The overall accuracies achieved by the different CNN architectures that have been used extensively for brain tumor classification are summarized in Figure 11. It shows that the majority of CNN models have achieved high performance for brain tumor classification tasks, in which transfer learning with ResNet, VGG, and GoogleNet showed more stable performance than other models, such as 3D CNN. Among the reviewed articles, five articles utilized 3D CNN for brain tumor classification, and the classification accuracy of those studies fluctuates wildly. The highest accuracy was 97.1%, achieved by Zhuge et al. [77], who trained a 3D CNN architecture with a dataset of 315 patients (210 HGG, 105 LGG). The lowest accuracy of 75% was obtained by Pei et al. [118], who used 398 brain MR image volumes for GBM vs. AST vs. OLI classification. In another study [117], the authors explored a 3D CNN model for OLI and AST classification using a very small dataset of 32 patients (16 OLI, 16 AST) and obtained a low accuracy of 80%. It seems that 3D CNN is a promising technique for realizing patient-wise diagnosis, and the accessibility of a large MRI dataset can hopefully improve the performance of 3D CNNs on brain tumor classification tasks.

### 5.4. The Effect of Pre-Processing and Data Augmentation Methods

**Figure 12:**
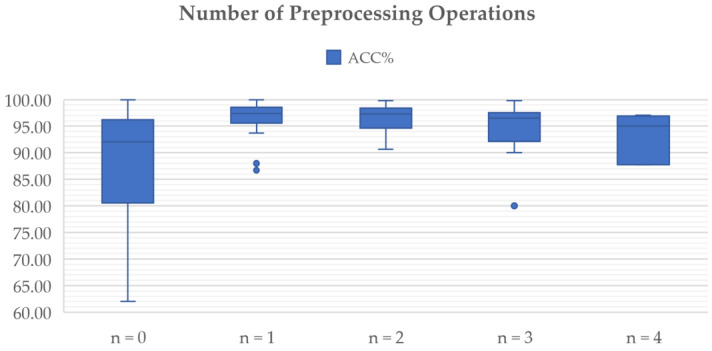
Classification accuracy by number of preprocessing operations.

Researchers have paid increasing amounts of attention to enhancing input image quality by conducting different preprocessing steps on brain MRI datasets before propagating them into CNN architectures. No studies have systematically tested the number and combination of operations that optimize classification accuracy. Figure 12 presents the overall accuracy obtained with different numbers of preprocessing operations. It shows that the studies that pre-processed input MR images collectively obtained higher classification accuracies than the studies that performed no preprocessing methods. However, it is not obvious that more steps led to better performance.

**Figure 13:**
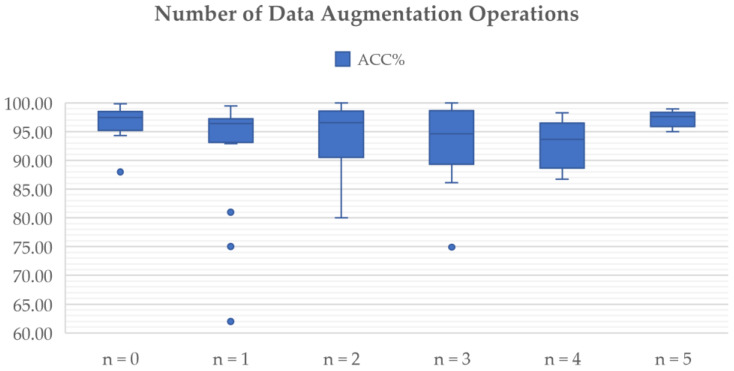
Classification accuracy by number of data augmentation operations.

As previously stated, data augmentation can create variations in the images that can improve the generalization capability of the models to new images, and different data augmentation techniques have been widely explored and applied to increase both the amount and the diversity of training data. Figure 13 illustrates the overall accuracy obtained with different numbers of data augmentation operations. It can be seen that studies that performed five data augmentation techniques achieved higher and more stable classification performance than the studies that performed fewer operations.

The accuracy data do not support the use of any single data augmentation method. It is interesting to ask whether data augmentation techniques were implemented specifically in those studies that lacked training data. However, on average, there is little difference between the 59 studies including or the 27 omitting a data augmentation step. On average, the former included 233 cases or 4743 images, and the latter included 269 cases or 7517 images. Curiously, the number of studies employing data augmentation has fallen as a proportion among those published in 2022, both compared to the total and compared to those using pre-processing methods.

**Figure 14:**
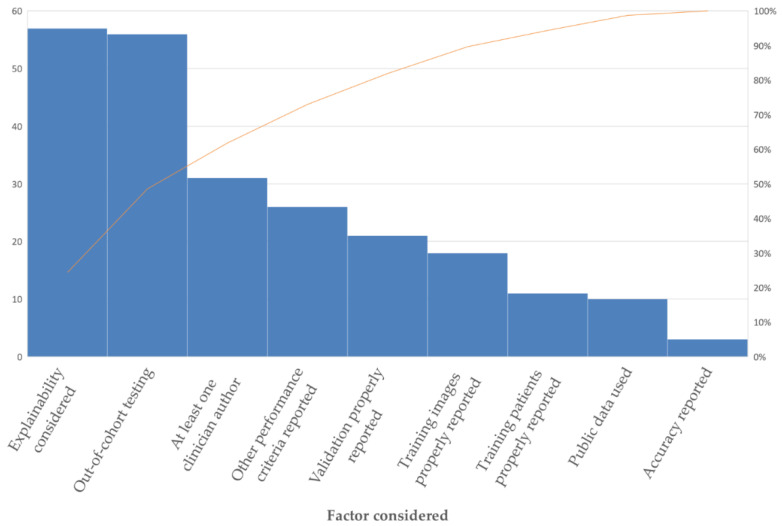
Histogram (left scale) and cumulative distribution (right scale) of factors not fully reported or considered in the studies reported in Table 4.

Figure 14 indicates the cumulative impact of factors that are not fully reported or considered in the studies reported in Table 4. Articles with multiple analyses for which factors differed were scored 1 (i.e., missing). Data are derived from Table 4, with the following exceptions: “Explainability considered” means that there was some analysis within the article on the information used to come to a diagnosis. Out-of-cohort testing occurred when CNN testing was performed on a cohort that was not used in the training/validation phase (i.e., different hospital or scanner). Author affiliations were derived from the author information in the DOI/CrossRef listed in the bibliography. An author was considered to have a clinical affiliation if their listed affiliations included a department of radiology, clinical neurology, neurosurgery, or oncology.

From the figure, the category other performance criteria performed means that performance criteria other than accuracy were reported. Validation was considered to be not properly reported if it was not performed or if the methods used in the validation step were not clearly described. Training patients/images properly reported means that the number of patients/images in each category used for training/validation is explicitly defined. Both factors are relevant as separate images from the same patient and are not fully independent. Public data used means that the data used are available to other researchers. In practice, all of the public data used were gathered in other studies, and no non-public data were made available by any of the studies identified.

### 5.5. The Effect of Other Factors

Beyond showing accuracy gains, the surveyed articles rarely examined their generalization capability and interpretability. Only very few studies [73,120] tested their classification models on an independent dataset, and only one study [122] investigated the Black-Box characteristic of CNN models for brain tumor classification to ensure that the model they obtained was looking in the correct place for decision-making rather than at noise or unrelated artifacts.

A limitation of this survey arises from the challenge of making comparisons in an objective manner between studies to analyze how each degrading factor affects the classification performance. One reason is that some studies worked on the same classification task but utilized different datasets, preprocessing methods, or classification techniques. Another reason lies in the variety of performance metrics reported. While accuracy was the most popular performance metric, it was not universally reported. Based on the difficulties encountered in the preparation of the present review, we suggest that at the very least, all deep learning studies for classification clearly report the classification accuracy of the models constructed and the numbers of images/subjects of each class used for training, validation, and testing purposes.

### 5.6. Future Directions

It is clear from the comparative analysis presented in Table 4b that CNN techniques and algorithms have great power and ability to handle medical MR data, but so far, but none of them are at the point of clinical usability. The challenges we have identified here must be appropriately addressed if CNN research is to be translated into clinic practice. This review has identified some common performance-degrading factors and potential solutions.

#### 5.6.1. The Training Data Problem

An exorbitant number of training cases are required to train a deep learning algorithm from scratch. With a limited number of training data, transfer learning with fine-tuning on pre-trained CNNs was demonstrated to yield better results for brain tumor classification than training such CNNs from scratch [45,116]. This is an efficient method for training networks when training data are expensive or difficult to collect in medical fields. In addition, high hardware requirements and long training times are also challenges that CNN-based CADx brain tumor classification systems face in clinical applications today. The continued development of state-of-the-art CNN architectures has resulted with a voracious appetite for computing power. Since the cost of training a deep learning model scales with the number of parameters and the amount of input data, this implies that computational requirements grow at the rate of at least the square of the number of training data [140]. With pre-trained models, transfer learning is also promising to address the difficulties caused by high hardware requirements and long training times when adopting CNN-based CADx systems for brain tumor classification in clinical practice. There are many issues related to optimizing transfer learning that remain to be studied.

#### 5.6.2. The Evaluation Problem

CADx systems are mainly used for educational and training purposes but not in clinical practice. Clinics still hesitate to use CADx-based systems. One reason for this is the lack of standardized methods for evaluating CADx systems in a realistic setting. The performance measures described in Section 4.2 are a useful and necessary baseline to compare algorithms, but they are all highly sensitive to the training set used, and more sophisticated tools are needed. It would be useful to define a pathway towards in-use performance evaluation, such as what was recently proposed for quantitative neuroradiology [141]. It is notable that many of the papers reviewed did not include any authors with a clinical background and that the image formats used to train the models were those typical of the AI research community (PNG) and not those of the radiology community (DICOM, NIfTI).

#### 5.6.3. Explainability and Trust

The Black-Box nature of deep CNNs has greatly limited their application outside of a research context. To trust systems powered by CNN models, clinicians need to know how they make predictions. However, among the articles surveyed, very few addressed this problem. The authors in [142] proposed a prototypical part network (ProtoPNet) that can highlight the image regions used for decision-making and can explain the reasoning process for the classification target by comparing the representative patches of the test image with the prototypes learned from a large number of data. To date, several studies have tested the explanation model proposed in [142] that was able to highlight image regions used for decision making in medical imaging fields, such as for mass lesion classification [143], lung disease detection [144,145], and Alzheimer’s diseases classification [146]. Future research in the brain tumor classification field will need to test how explainable models influence the attitudes and decision-making processes of radiologists or other clinicians.

The lack of physician training on how to interact with CADx systems and how to interpret their results to make diagnostic decisions is a separate but related technical challenge that can reduce the performance of CADx systems in practice, something that is not addressed in any of the papers included in the review. A greater role for physicians in the research process may bring benefits both in terms of the relevance of research projects and the acceptance of their results.

In summary, the future of CNN-based brain tumor classification studies is very promising and focusing on the right direction with references to the challenges mentioned above would advance these studies from research labs to hospitals. We believe that our review provides researchers in the biomedical and machine learning communities with indicators for useful future directions for this purpose.

## 6. Conclusions

CADx systems may play an important role in assisting physicians in making decisions. This paper surveyed 83 articles that adopted CNNs for brain MRI classification and analyzed the challenges and barriers that CNN-based CADx brain tumor classification systems face today in clinical application and development. A detailed analysis of the potential factors that affect classification accuracy is provided in this study. From the comparative analysis in Table 4b, it is clear that CNN techniques and algorithms have great power and ability to handle medical MR data. However, many of the CNN classification models that have been developed so far still are still lacking in one way or another in terms of clinical application and development. Research oriented towards appropriately addressing the challenges noted here can help drive the translation of CNN research into clinical practice for brain tumor classification. In this review, some performance degrading factors and their solutions are also discussed to provide researchers in the biomedical and machine learning communities with indicators for developing optimized CADx systems for brain tumor classification.

## Figures and Tables

**Table 1:** The search queries used to interrogate the PubMed and Scopus databases.

PubMed /Scopus	(deep learning OR deep model OR artificial intelligence OR artificial neural network OR autoencoder OR generative adversarial network) OR convolutional OR (neural network) OR neural network OR deep model OR convolutional)	AND
	(brain tumor OR glioma OR brain cancer OR glioblastoma OR astrocytoma OR oligodendroglioma OR ependymoma)	AND
	(classification OR grading OR classify)	AND
	(MRI OR Magnetic Resonance OR MR images OR radiographic OR radiology)	IN
	Title/Abstract	

**Table 3:** The imaging configurations and main clinical distinctions of T_1_w, T_2_w, ceT_1_w, and FLAIR.

Sequence	Sequence Characteristics	Main Clinical Distinctions	Example *
T_1_w	Uses short TR and TE [64]	Lower signal for a higher water content [66], such as in edema, tumor, inflammation, infection, or chronic hemorrhage [66] Higher signal for fat [66] Higher signal for subacute hemorrhage [66]	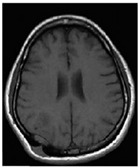
T_2_w	Uses long TR and TE [64]	Higher signal for a higher water content, such as in edema, tumor, infarction, inflammation, infection, or subdural collection [66] Lower signal for fat [66] Lower signal for fibrous tissue [66]	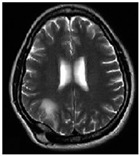
ceT_1_w	Uses the same TR and TE as T_1_w; employs contrast agents [64]	Higher signal for areas of breakdown in the blood–brain barrier that indicate induced inflammation [65]	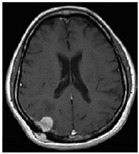
FLAIR	Uses very long TR and TE; the inversion time nulls the signal from fluid [67]	Highest signal for abnormalities [65] Highest signal for gray matter [67] Lower signal for cerebrospinal fluid [67]	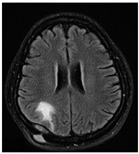

* Pictures from [68]. TR, repetition time. TE, echo time.

## Data Availability

Not applicable.

## Supplementary Materials

The following supporting information can be downloaded at: https://www.mdpi.com/article/10.3390/diagnostics12081850/s1, Table S1: Article Screening Recording.
